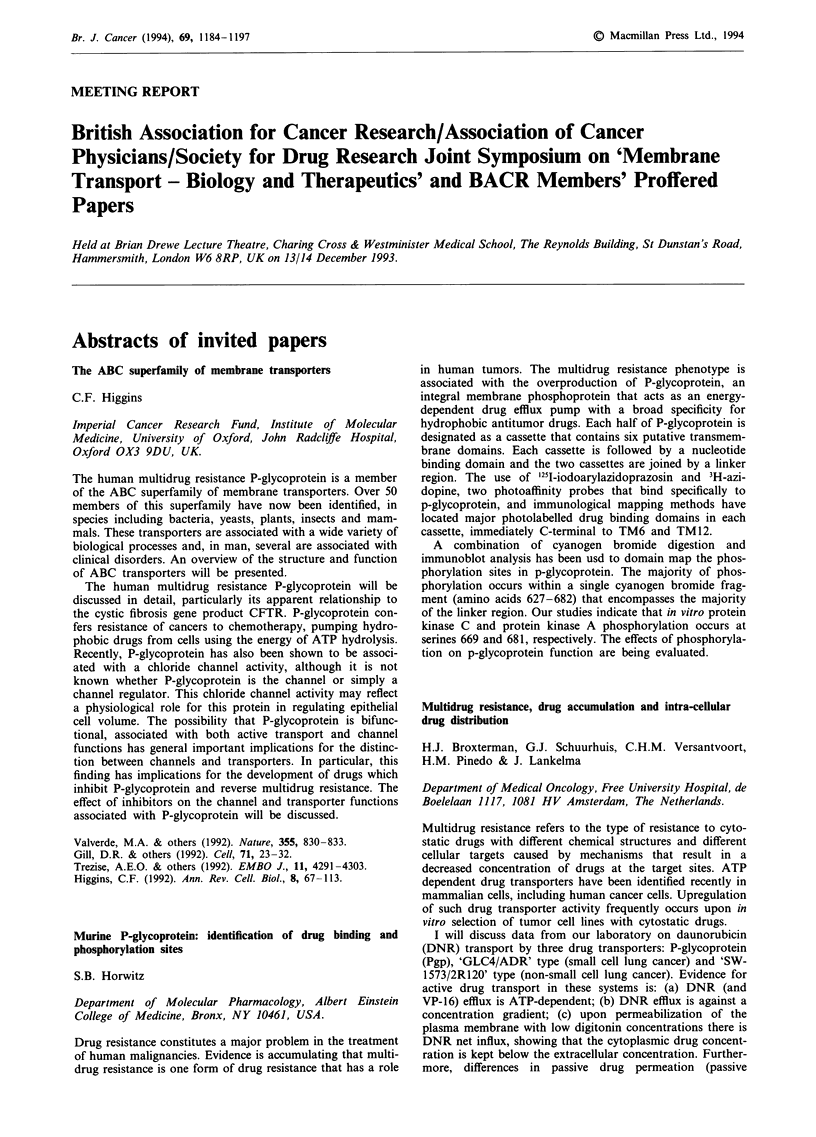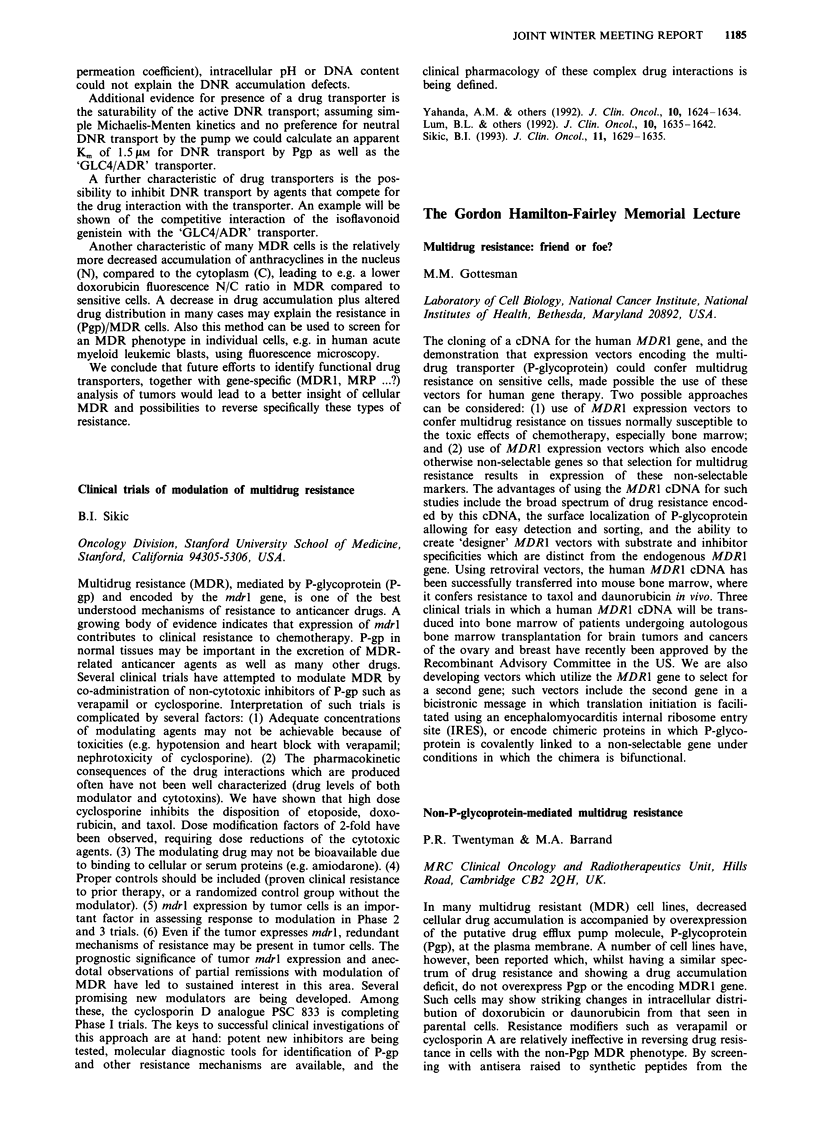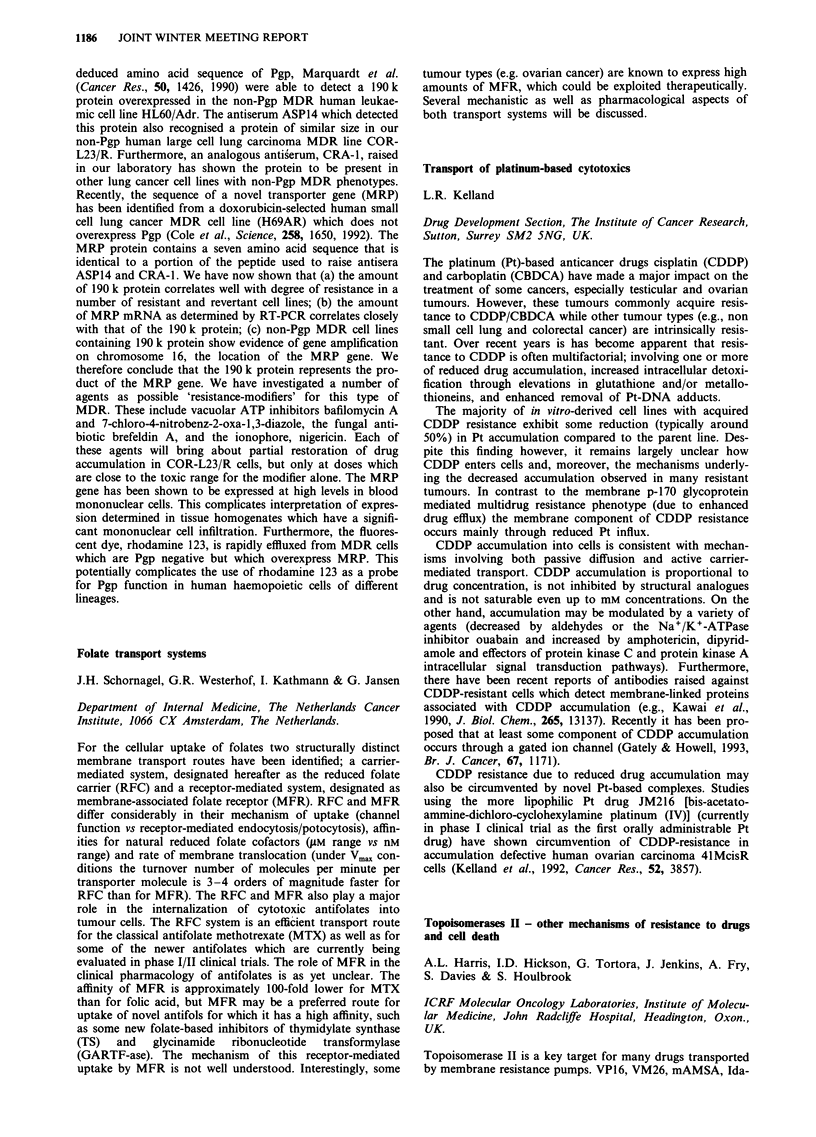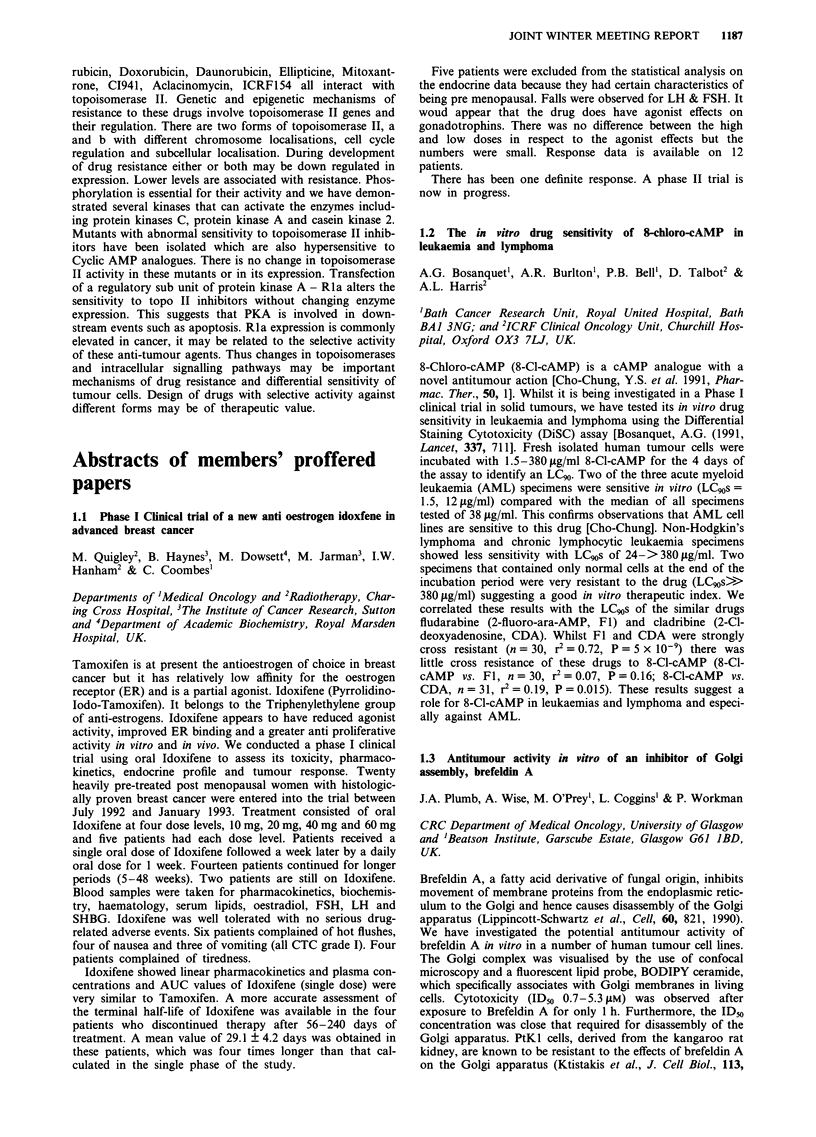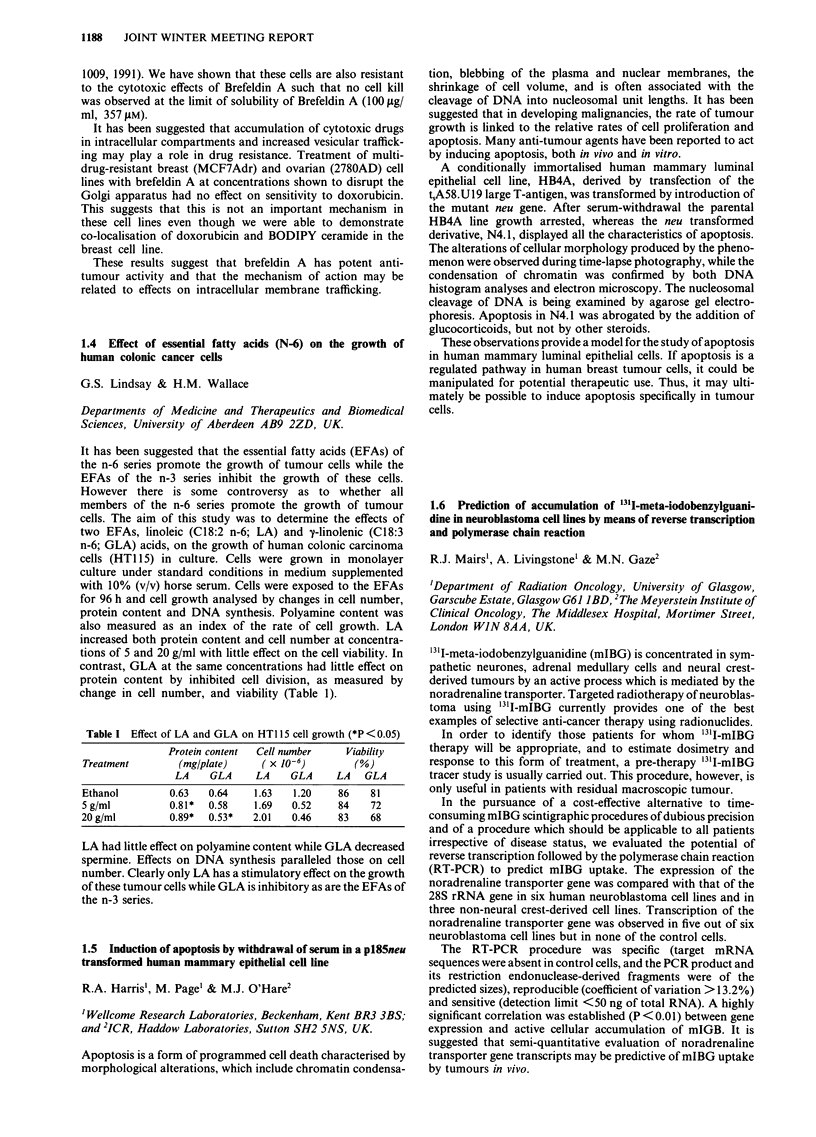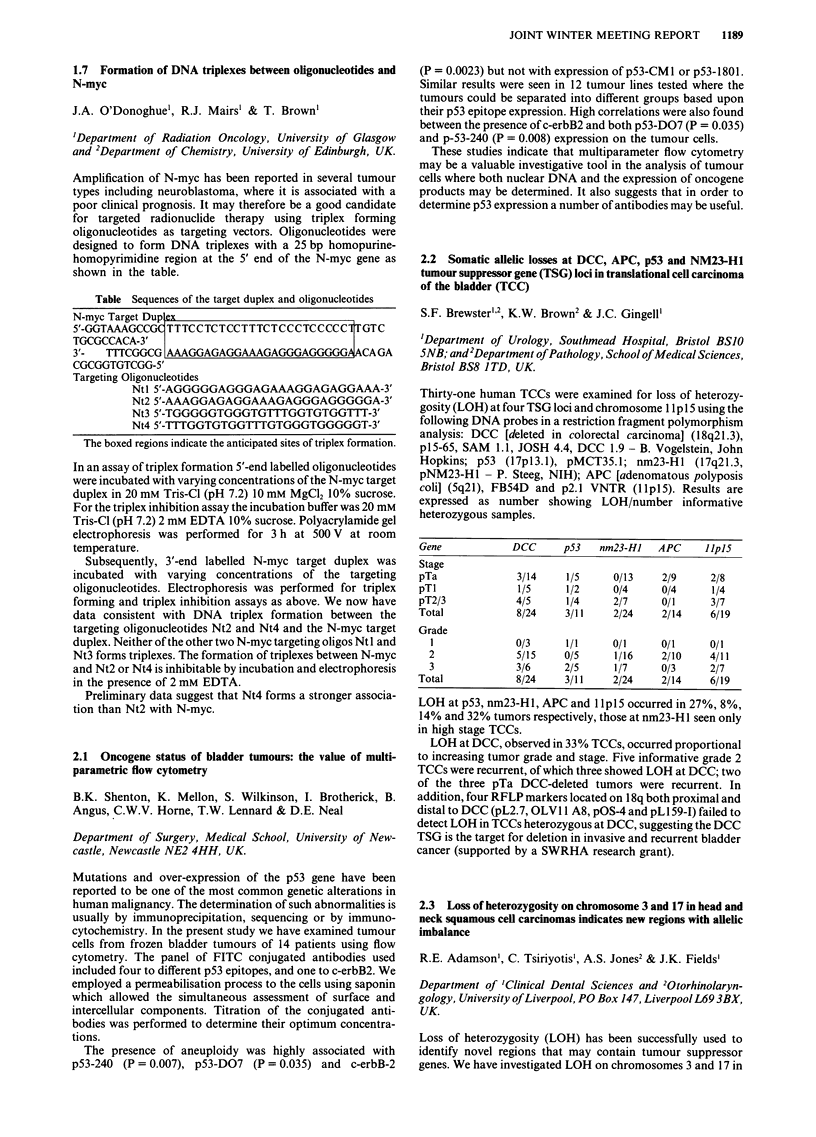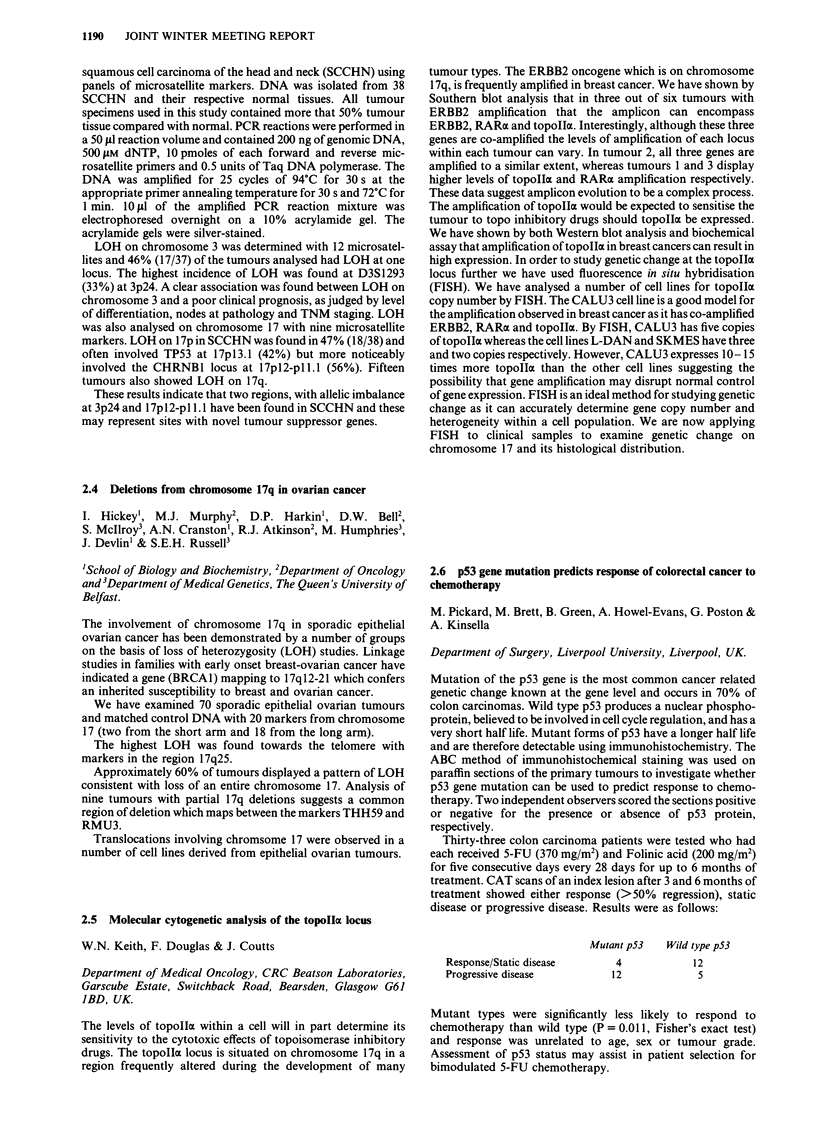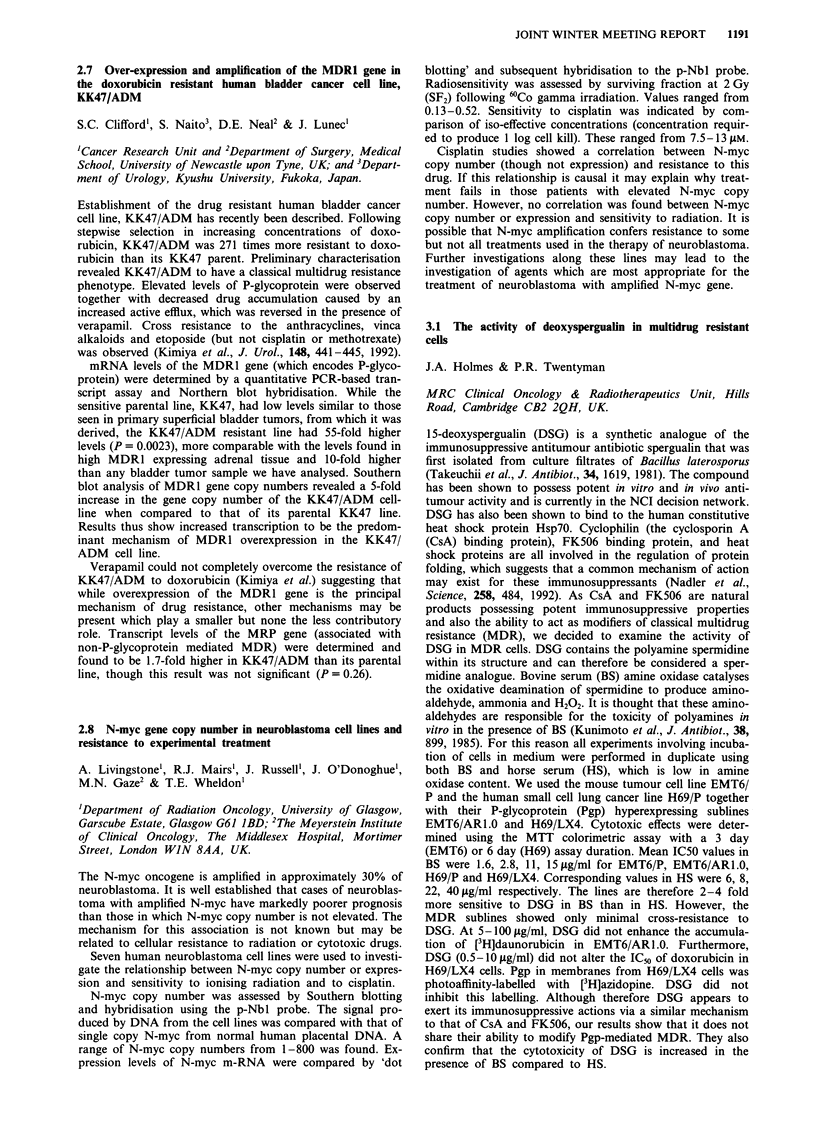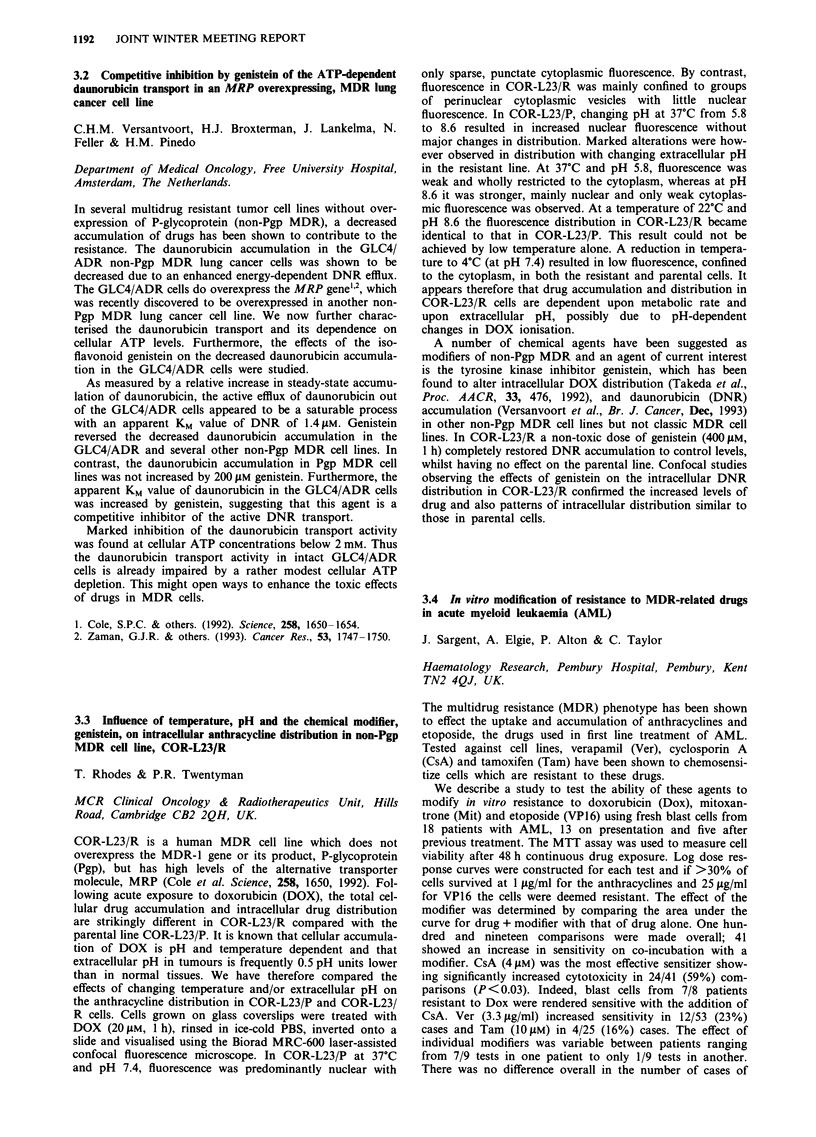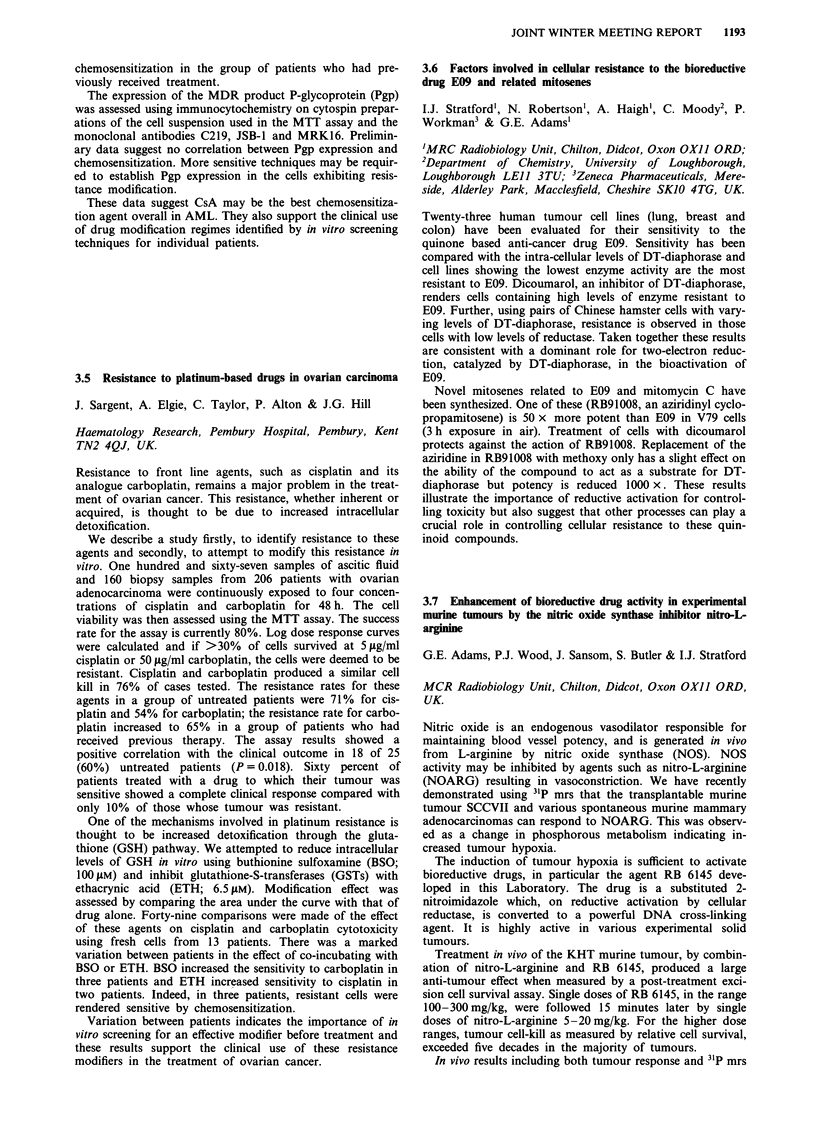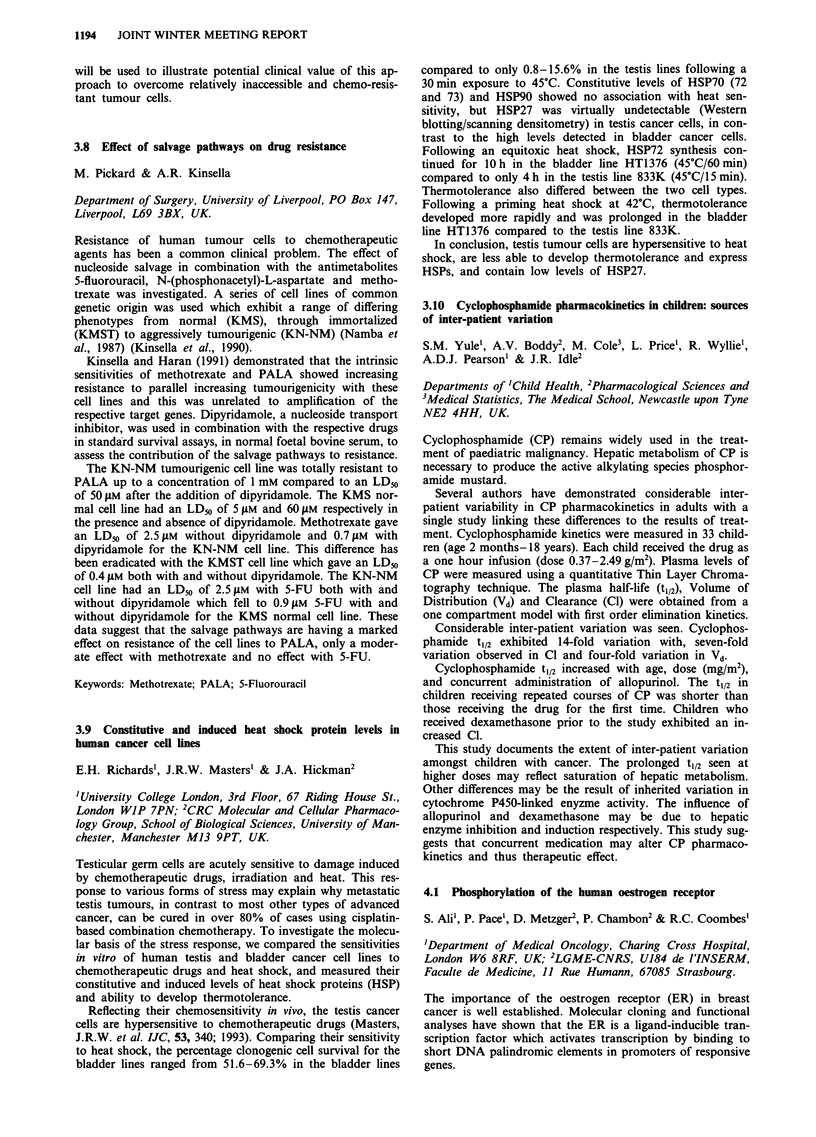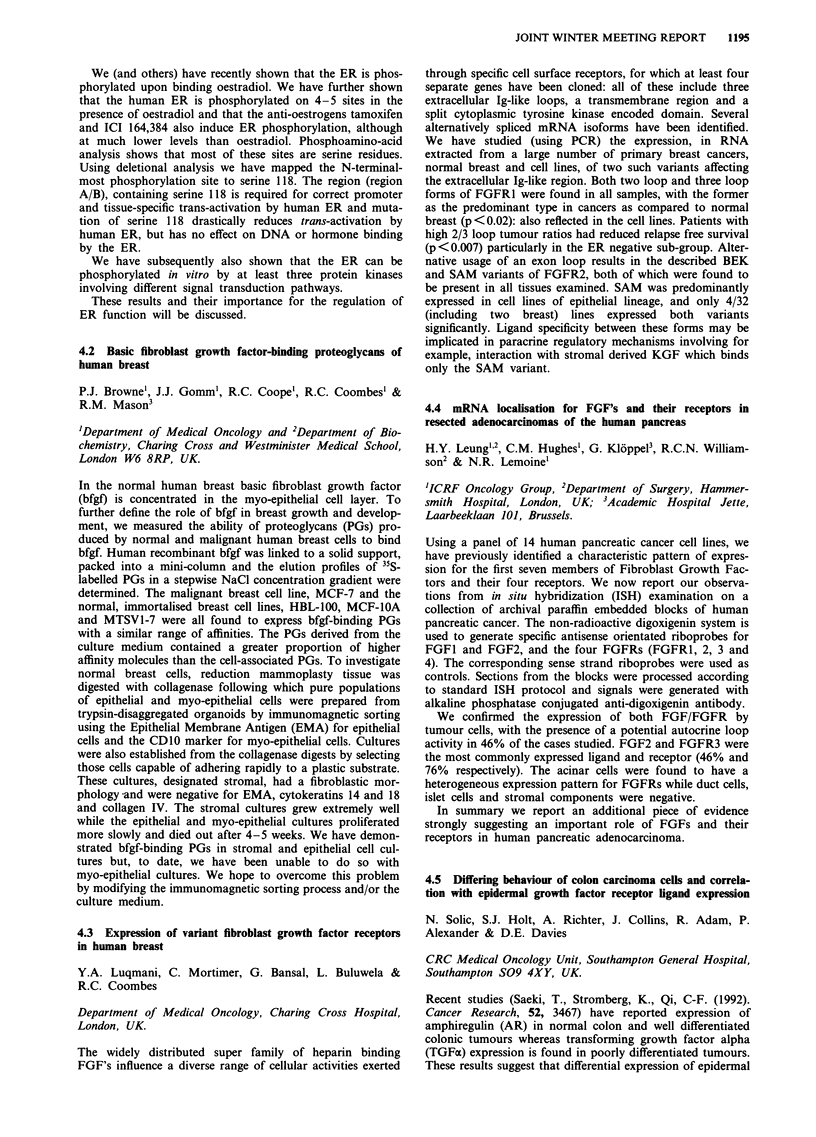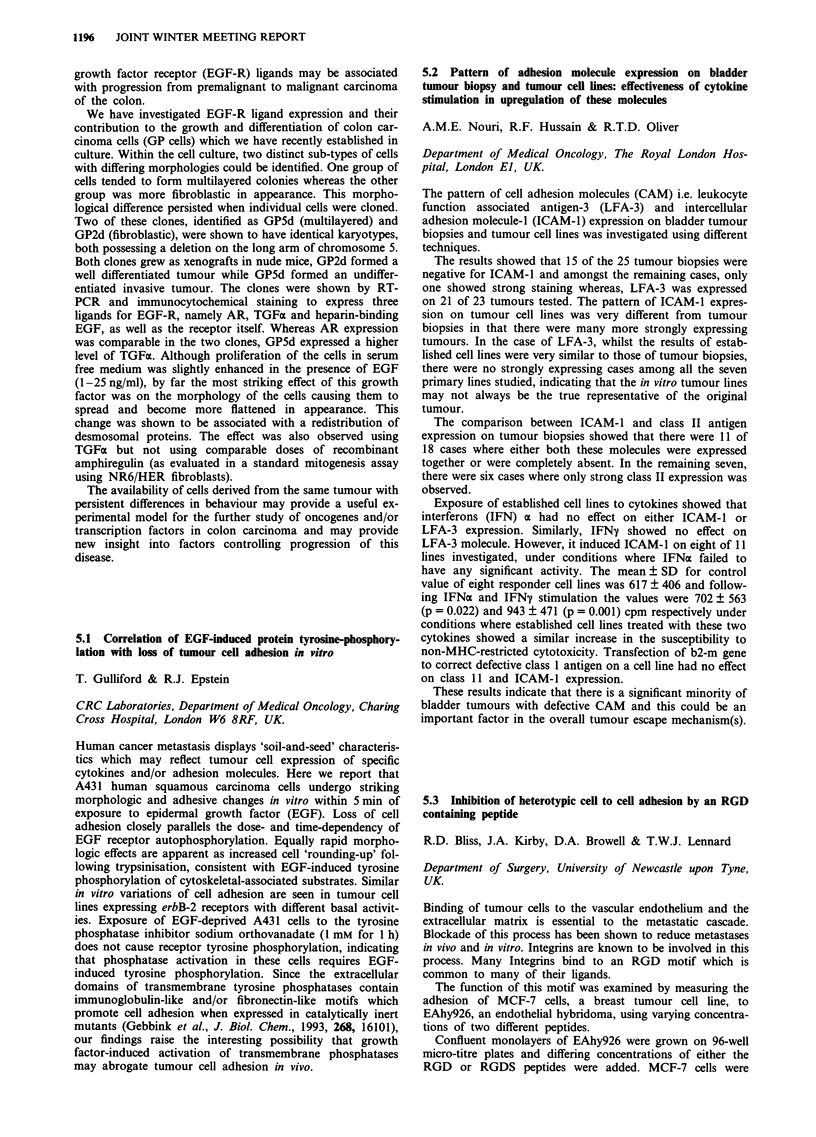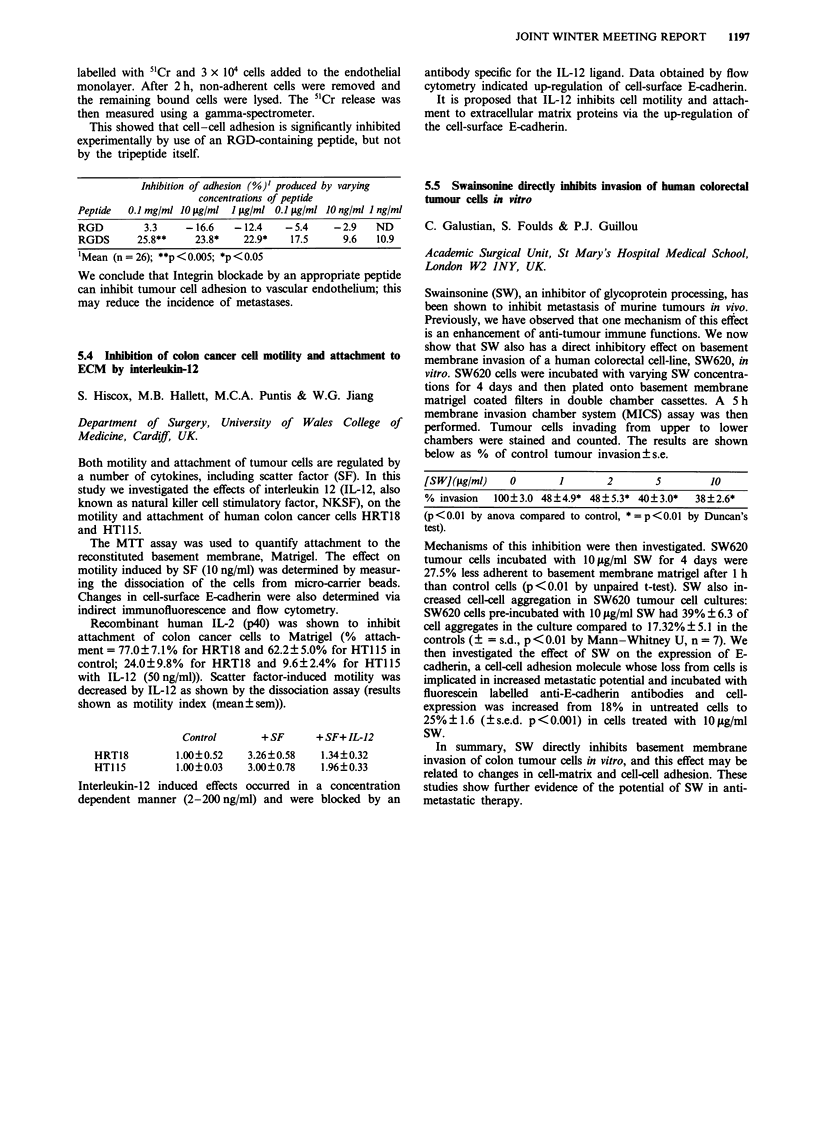# British Association for Cancer Research/'Association of Cancer Physicians/Society for Drug Research Joint Symposium on `Membrane Transport - Biology and Therapeutics' and BACR Members' Proffered Papers

**Published:** 1994-06

**Authors:** 


Br. J. Cancer (1994), 69, 1184–1197 © Macmillan Press Ltd., 1994

MEETING REPORT

### British Association for Cancer Research/Association of Cancer Physicians/Society for Drug Research Joint Symposium on 'Membrane Transport – Biology and Therapeutics' and BACR Members' Proffered Papers

*Held at Brian Drewe Lecture Theatre, Charing Cross & Westminister Medical School, The Reynolds Building, St Dunstan's Road, Hammersmith, London W6 8RP, UK on 13/14 December 1993.*

### Abstracts of invited papers

### The ABC superfamily of membrane transporters

C.F. Higgins

*Imperial Cancer Research Fund, Institute of Molecular Medicine, University of Oxford, John Radcliffe Hospital, Oxford OX3 9DU, UK.*

The human multidrug resistance P-glycoprotein is a member of the ABC superfamily of membrane transporters. Over 50 members of this superfamily have now been identified, in species including bacteria, yeasts, plants, insects and mammals. These transporters are associated with a wide variety of biological processes and, in man, several are associated with clinical disorders. An overview of the structure and function of ABC transporters will be presented.

The human multidrug resistance P-glycoprotein will be discussed in detail, particularly its apparent relationship to the cystic fibrosis gene product CFTR. P-glycoprotein confers resistance of cancers to chemotherapy, pumping hydrophobic drugs from cells using the energy of ATP hydrolysis. Recently, P-glycoprotein has also been shown to be associated with a chloride channel activity, although it is not known whether P-glycoprotein is the channel or simply a channel regulator. This chloride channel activity may reflect a physiological role for this protein in regulating epithelial cell volume. The possibility that P-glycoprotein is bifunctional, associated with both active transport and channel functions has general important implications for the distinction between channels and transporters. In particular, this finding has implications for the development of drugs which inhibit P-glycoprotein and reverse multidrug resistance. The effect of inhibitors on the channel and transporter functions associated with P-glycoprotein will be discussed.

Valverde, M.A. & others (1992). *Nature*, **355**, 830–833.
Gill, D.R. & others (1992). *Cell*, **71**, 23–32.
Trezise, A.E.O. & others (1992). *EMBO J.*, **11**, 4291–4303.
Higgins, C.F. (1992). *Ann. Rev. Cell. Biol.*, **8**, 67–113.

### Murine P-glycoprotein: identification of drug binding and phosphorylation sites

S.B. Horwitz

*Department of Molecular Pharmacology, Albert Einstein College of Medicine, Bronx, NY 10461, USA.*

Drug resistance constitutes a major problem in the treatment of human malignancies. Evidence is accumulating that multidrug resistance is one form of drug resistance that has a role in human tumors. The multidrug resistance phenotype is associated with the overproduction of P-glycoprotein, an integral membrane phosphoprotein that acts as an energy-dependent drug efflux pump with a broad specificity for hydrophobic antitumor drugs. Each half of P-glycoprotein is designated as a cassette that contains six putative transmembrane domains. Each cassette is followed by a nucleotide binding domain and the two cassettes are joined by a linker region. The use of $^{125}$I-iodoarylazidoprazosin and $^{3}$H-azidopine, two photoaffinity probes that bind specifically to p-glycoprotein, and immunological mapping methods have located major photolabelled drug binding domains in each cassette, immediately C-terminal to TM6 and TM12.

A combination of cyanogen bromide digestion and immunoblot analysis has been usd to domain map the phosphorylation sites in p-glycoprotein. The majority of phosphorylation occurs within a single cyanogen bromide fragment (amino acids 627–682) that encompasses the majority of the linker region. Our studies indicate that *in vitro* protein kinase C and protein kinase A phosphorylation occurs at serines 669 and 681, respectively. The effects of phosphorylation on p-glycoprotein function are being evaluated.

### Multidrug resistance, drug accumulation and intra-cellular drug distribution

H.J. Broxterman, G.J. Schuurhuis, C.H.M. Versantvoort, H.M. Pinedo & J. Lankelma

*Department of Medical Oncology, Free University Hospital, de Boelelaan 1117, 1081 HV Amsterdam, The Netherlands.*

Multidrug resistance refers to the type of resistance to cytostatic drugs with different chemical structures and different cellular targets caused by mechanisms that result in a decreased concentration of drugs at the target sites. ATP dependent drug transporters have been identified recently in mammalian cells, including human cancer cells. Upregulation of such drug transporter activity frequently occurs upon *in vitro* selection of tumor cell lines with cytostatic drugs.

I will discuss data from our laboratory on daunorubicin (DNR) transport by three drug transporters: P-glycoprotein (Pgp), 'GLC4/ADR' type (small cell lung cancer) and 'SW-1573/2R120' type (non-small cell lung cancer). Evidence for active drug transport in these systems is: (a) DNR (and VP-16) efflux is ATP-dependent; (b) DNR efflux is against a concentration gradient; (c) upon permeabilization of the plasma membrane with low digitonin concentrations there is DNR net influx, showing that the cytoplasmic drug concentration is kept below the extracellular concentration. Furthermore, differences in passive drug permeation (passive permeation coefficient), intracellular pH or DNA content could not explain the DNR accumulation defects.

Additional evidence for presence of a drug transporter is the saturability of the active DNR transport; assuming simple Michaelis-Menten kinetics and no preference for neutral DNR transport by the pump we could calculate an apparent $K_m$ of 1.5 μM for DNR transport by Pgp as well as the 'GLC4/ADR' transporter.

A further characteristic of drug transporters is the possibility to inhibit DNR transport by agents that compete for the drug interaction with the transporter. An example will be shown of the competitive interaction of the isoflavonoid genistein with the 'GLC4/ADR' transporter.

Another characteristic of many MDR cells is the relatively more decreased accumulation of anthracyclines in the nucleus (N), compared to the cytoplasm (C), leading to e.g. a lower doxorubicin fluorescence N/C ratio in MDR compared to sensitive cells. A decrease in drug accumulation plus altered drug distribution in many cases may explain the resistance in (Pgp)/MDR cells. Also this method can be used to screen for an MDR phenotype in individual cells, e.g. in human acute myeloid leukemic blasts, using fluorescence microscopy.

We conclude that future efforts to identify functional drug transporters, together with gene-specific (MDR1, MRP ...?) analysis of tumors would lead to a better insight of cellular MDR and possibilities to reverse specifically these types of resistance.

### Clinical trials of modulation of multidrug resistance

B.I. Sikic

*Oncology Division, Stanford University School of Medicine, Stanford, California 94305-5306, USA.*

Multidrug resistance (MDR), mediated by P-glycoprotein (P-gp) and encoded by the *mdr*1 gene, is one of the best understood mechanisms of resistance to anticancer drugs. A growing body of evidence indicates that expression of *mdr*1 contributes to clinical resistance to chemotherapy. P-gp in normal tissues may be important in the excretion of MDR-related anticancer agents as well as many other drugs. Several clinical trials have attempted to modulate MDR by co-administration of non-cytotoxic inhibitors of P-gp such as verapamil or cyclosporine. Interpretation of such trials is complicated by several factors: (1) Adequate concentrations of modulating agents may not be achievable because of toxicities (e.g. hypotension and heart block with verapamil; nephrotoxicity of cyclosporine). (2) The pharmacokinetic consequences of the drug interactions which are produced often have not been well characterized (drug levels of both modulator and cytotoxins). We have shown that high dose cyclosporine inhibits the disposition of etoposide, doxorubicin, and taxol. Dose modification factors of 2-fold have been observed, requiring dose reductions of the cytotoxic agents. (3) The modulating drug may not be bioavailable due to binding to cellular or serum proteins (e.g. amiodarone). (4) Proper controls should be included (proven clinical resistance to prior therapy, or a randomized control group without the modulator). (5) *mdr*1 expression by tumor cells is an important factor in assessing response to modulation in Phase 2 and 3 trials. (6) Even if the tumor expresses *mdr*1, redundant mechanisms of resistance may be present in tumor cells. The prognostic significance of tumor *mdr*1 expression and anecdotal observations of partial remissions with modulation of MDR have led to sustained interest in this area. Several promising new modulators are being developed. Among these, the cyclosporin D analogue PSC 833 is completing Phase I trials. The keys to successful clinical investigations of this approach are at hand: potent new inhibitors are being tested, molecular diagnostic tools for identification of P-gp and other resistance mechanisms are available, and the clinical pharmacology of these complex drug interactions is being defined.

Yahanda, A.M. & others (1992). *J. Clin. Oncol.*, **10**, 1624–1634.
Lum, B.L. & others (1992). *J. Clin. Oncol.*, **10**, 1635–1642.
Sikic, B.I. (1993). *J. Clin. Oncol.*, **11**, 1629–1635.

### The Gordon Hamilton-Fairley Memorial Lecture

### Multidrug resistance: friend or foe?

M.M. Gottesman

*Laboratory of Cell Biology, National Cancer Institute, National Institutes of Health, Bethesda, Maryland 20892, USA.*

The cloning of a cDNA for the human *MDR*1 gene, and the demonstration that expression vectors encoding the multidrug transporter (P-glycoprotein) could confer multidrug resistance on sensitive cells, made possible the use of these vectors for human gene therapy. Two possible approaches can be considered: (1) use of *MDR*1 expression vectors to confer multidrug resistance on tissues normally susceptible to the toxic effects of chemotherapy, especially bone marrow; and (2) use of *MDR*1 expression vectors which also encode otherwise non-selectable genes so that selection for multidrug resistance results in expression of these non-selectable markers. The advantages of using the *MDR*1 cDNA for such studies include the broad spectrum of drug resistance encoded by this cDNA, the surface localization of P-glycoprotein allowing for easy detection and sorting, and the ability to create 'designer' *MDR*1 vectors with substrate and inhibitor specificities which are distinct from the endogenous *MDR*1 gene. Using retroviral vectors, the human *MDR*1 cDNA has been successfully transferred into mouse bone marrow, where it confers resistance to taxol and daunorubicin *in vivo*. Three clinical trials in which a human *MDR*1 cDNA will be transduced into bone marrow of patients undergoing autologous bone marrow transplantation for brain tumors and cancers of the ovary and breast have recently been approved by the Recombinant Advisory Committee in the US. We are also developing vectors which utilize the *MDR*1 gene to select for a second gene; such vectors include the second gene in a bicistronic message in which translation initiation is facilitated using an encephalomyocarditis internal ribosome entry site (IRES), or encode chimeric proteins in which P-glycoprotein is covalently linked to a non-selectable gene under conditions in which the chimera is bifunctional.

### Non-P-glycoprotein-mediated multidrug resistance

P.R. Twentyman & M.A. Barrand

*MRC Clinical Oncology and Radiotherapeutics Unit, Hills Road, Cambridge CB2 2QH, UK.*

In many multidrug resistant (MDR) cell lines, decreased cellular drug accumulation is accompanied by overexpression of the putative drug efflux pump molecule, P-glycoprotein (Pgp), at the plasma membrane. A number of cell lines have, however, been reported which, whilst having a similar spectrum of drug resistance and showing a drug accumulation deficit, do not overexpress Pgp or the encoding MDR1 gene. Such cells may show striking changes in intracellular distribution of doxorubicin or daunorubicin from that seen in parental cells. Resistance modifiers such as verapamil or cyclosporin A are relatively ineffective in reversing drug resistance in cells with the non-Pgp MDR phenotype. By screening with antisera raised to synthetic peptides from the deduced amino acid sequence of Pgp, Marquardt *et al.* (*Cancer Res.*, **50,** 1426, 1990) were able to detect a 190 k protein overexpressed in the non-Pgp MDR human leukaemic cell line HL60/Adr. The antiserum ASP14 which detected this protein also recognised a protein of similar size in our non-Pgp human large cell lung carcinoma MDR line COR-L23/R. Furthermore, an analogous antiserum, CRA-1, raised in our laboratory has shown the protein to be present in other lung cancer cell lines with non-Pgp MDR phenotypes. Recently, the sequence of a novel transporter gene (MRP) has been identified from a doxorubicin-selected human small cell lung cancer MDR cell line (H69AR) which does not overexpress Pgp (Cole *et al.*, *Science*, **258,** 1650, 1992). The MRP protein contains a seven amino acid sequence that is identical to a portion of the peptide used to raise antisera ASP14 and CRA-1. We have now shown that (a) the amount of 190 k protein correlates well with degree of resistance in a number of resistant and revertant cell lines; (b) the amount of MRP mRNA as determined by RT-PCR correlates closely with that of the 190 k protein; (c) non-Pgp MDR cell lines containing 190 k protein show evidence of gene amplification on chromosome 16, the location of the MRP gene. We therefore conclude that the 190 k protein represents the product of the MRP gene. We have investigated a number of agents as possible 'resistance-modifiers' for this type of MDR. These include vacuolar ATP inhibitors bafilomycin A and 7-chloro-4-nitrobenz-2-oxa-1,3-diazole, the fungal antibiotic brefeldin A, and the ionophore, nigericin. Each of these agents will bring about partial restoration of drug accumulation in COR-L23/R cells, but only at doses which are close to the toxic range for the modifier alone. The MRP gene has been shown to be expressed at high levels in blood mononuclear cells. This complicates interpretation of expression determined in tissue homogenates which have a significant mononuclear cell infiltration. Furthermore, the fluorescent dye, rhodamine 123, is rapidly effluxed from MDR cells which are Pgp negative but which overexpress MRP. This potentially complicates the use of rhodamine 123 as a probe for Pgp function in human haemopoietic cells of different lineages.

### Folate transport systems

J.H. Schornagel, G.R. Westerhof, I. Kathmann & G. Jansen

*Department of Internal Medicine, The Netherlands Cancer Institute, 1066 CX Amsterdam, The Netherlands.*

For the cellular uptake of folates two structurally distinct membrane transport routes have been identified; a carrier-mediated system, designated hereafter as the reduced folate carrier (RFC) and a receptor-mediated system, designated as membrane-associated folate receptor (MFR). RFC and MFR differ considerably in their mechanism of uptake (channel function *vs* receptor-mediated endocytosis/potocytosis), affinities for natural reduced folate cofactors ($\mu$M range *vs* nM range) and rate of membrane translocation (under $V_{max}$ conditions the turnover number of molecules per minute per transporter molecule is 3–4 orders of magnitude faster for RFC than for MFR). The RFC and MFR also play a major role in the internalization of cytotoxic antifolates into tumour cells. The RFC system is an efficient transport route for the classical antifolate methotrexate (MTX) as well as for some of the newer antifolates which are currently being evaluated in phase I/II clinical trials. The role of MFR in the clinical pharmacology of antifolates is as yet unclear. The affinity of MFR is approximately 100-fold lower for MTX than for folic acid, but MFR may be a preferred route for uptake of novel antifols for which it has a high affinity, such as some new folate-based inhibitors of thymidylate synthase (TS) and glycinamide ribonucleotide transformylase (GARTF-ase). The mechanism of this receptor-mediated uptake by MFR is not well understood. Interestingly, some tumour types (e.g. ovarian cancer) are known to express high amounts of MFR, which could be exploited therapeutically. Several mechanistic as well as pharmacological aspects of both transport systems will be discussed.

### Transport of platinum-based cytotoxics

L.R. Kelland

*Drug Development Section, The Institute of Cancer Research, Sutton, Surrey SM2 5NG, UK.*

The platinum (Pt)-based anticancer drugs cisplatin (CDDP) and carboplatin (CBDCA) have made a major impact on the treatment of some cancers, especially testicular and ovarian tumours. However, these tumours commonly acquire resistance to CDDP/CBDCA while other tumour types (e.g., non small cell lung and colorectal cancer) are intrinsically resistant. Over recent years is has become apparent that resistance to CDDP is often multifactorial; involving one or more of reduced drug accumulation, increased intracellular detoxification through elevations in glutathione and/or metallothioneins, and enhanced removal of Pt-DNA adducts.

The majority of *in vitro*-derived cell lines with acquired CDDP resistance exhibit some reduction (typically around 50%) in Pt accumulation compared to the parent line. Despite this finding however, it remains largely unclear how CDDP enters cells and, moreover, the mechanisms underlying the decreased accumulation observed in many resistant tumours. In contrast to the membrane p-170 glycoprotein mediated multidrug resistance phenotype (due to enhanced drug efflux) the membrane component of CDDP resistance occurs mainly through reduced Pt influx.

CDDP accumulation into cells is consistent with mechanisms involving both passive diffusion and active carrier-mediated transport. CDDP accumulation is proportional to drug concentration, is not inhibited by structural analogues and is not saturable even up to mM concentrations. On the other hand, accumulation may be modulated by a variety of agents (decreased by aldehydes or the $Na^+/K^+$-ATPase inhibitor ouabain and increased by amphotericin, dipyridamole and effectors of protein kinase C and protein kinase A intracellular signal transduction pathways). Furthermore, there have been recent reports of antibodies raised against CDDP-resistant cells which detect membrane-linked proteins associated with CDDP accumulation (e.g., Kawai *et al.*, 1990, *J. Biol. Chem.*, **265,** 13137). Recently it has been proposed that at least some component of CDDP accumulation occurs through a gated ion channel (Gately & Howell, 1993, *Br. J. Cancer*, **67,** 1171).

CDDP resistance due to reduced drug accumulation may also be circumvented by novel Pt-based complexes. Studies using the more lipophilic Pt drug JM216 [bis-acetato-ammine-dichloro-cyclohexylamine platinum (IV)] (currently in phase I clinical trial as the first orally administrable Pt drug) have shown circumvention of CDDP-resistance in accumulation defective human ovarian carcinoma 41McisR cells (Kelland *et al.*, 1992, *Cancer Res.*, **52,** 3857).

### Topoisomerases II – other mechanisms of resistance to drugs and cell death

A.L. Harris, I.D. Hickson, G. Tortora, J. Jenkins, A. Fry, S. Davies & S. Houlbrook

*ICRF Molecular Oncology Laboratories, Institute of Molecular Medicine, John Radcliffe Hospital, Headington, Oxon., UK.*

Topoisomerase II is a key target for many drugs transported by membrane resistance pumps. VP16, VM26, mAMSA, Idarubicin, Doxorubicin, Daunorubicin, Ellipticine, Mitoxantrone, CI941, Aclacinomycin, ICRF154 all interact with topoisomerase II. Genetic and epigenetic mechanisms of resistance to these drugs involve topoisomerase II genes and their regulation. There are two forms of topoisomerase II, a and b with different chromosome localisations, cell cycle regulation and subcellular localisation. During development of drug resistance either or both may be down regulated in expression. Lower levels are associated with resistance. Phosphorylation is essential for their activity and we have demonstrated several kinases that can activate the enzymes including protein kinases C, protein kinase A and casein kinase 2. Mutants with abnormal sensitivity to topoisomerase II inhibitors have been isolated which are also hypersensitive to Cyclic AMP analogues. There is no change in topoisomerase II activity in these mutants or in its expression. Transfection of a regulatory sub unit of protein kinase A – R1a alters the sensitivity to topo II inhibitors without changing enzyme expression. This suggests that PKA is involved in downstream events such as apoptosis. R1a expression is commonly elevated in cancer, it may be related to the selective activity of these anti-tumour agents. Thus changes in topoisomerases and intracellular signalling pathways may be important mechanisms of drug resistance and differential sensitivity of tumour cells. Design of drugs with selective activity against different forms may be of therapeutic value.

### Abstracts of members' proffered papers

### 1.1 Phase I Clinical trial of a new anti oestrogen idoxfene in advanced breast cancer

M. Quigley[2], B. Haynes[3], M. Dowsett[4], M. Jarman[3], I.W. Hanham[2] & C. Coombes[1]

*Departments of [1]Medical Oncology and [2]Radiotherapy, Charing Cross Hospital, [3]The Institute of Cancer Research, Sutton and [4]Department of Academic Biochemistry, Royal Marsden Hospital, UK.*

Tamoxifen is at present the antioestrogen of choice in breast cancer but it has relatively low affinity for the oestrogen receptor (ER) and is a partial agonist. Idoxifene (Pyrrolidino-Iodo-Tamoxifen). It belongs to the Triphenylethylene group of anti-estrogens. Idoxifene appears to have reduced agonist activity, improved ER binding and a greater anti proliferative activity *in vitro* and *in vivo*. We conducted a phase I clinical trial using oral Idoxifene to assess its toxicity, pharmacokinetics, endocrine profile and tumour response. Twenty heavily pre-treated post menopausal women with histologically proven breast cancer were entered into the trial between July 1992 and January 1993. Treatment consisted of oral Idoxifene at four dose levels, 10 mg, 20 mg, 40 mg and 60 mg and five patients had each dose level. Patients received a single oral dose of Idoxifene followed a week later by a daily oral dose for 1 week. Fourteen patients continued for longer periods (5–48 weeks). Two patients are still on Idoxifene. Blood samples were taken for pharmacokinetics, biochemistry, haematology, serum lipids, oestradiol, FSH, LH and SHBG. Idoxifene was well tolerated with no serious drug-related adverse events. Six patients complained of hot flushes, four of nausea and three of vomiting (all CTC grade I). Four patients complained of tiredness.

Idoxifene showed linear pharmacokinetics and plasma concentrations and AUC values of Idoxifene (single dose) were very similar to Tamoxifen. A more accurate assessment of the terminal half-life of Idoxifene was available in the four patients who discontinued therapy after 56–240 days of treatment. A mean value of 29.1 ± 4.2 days was obtained in these patients, which was four times longer than that calculated in the single phase of the study.

Five patients were excluded from the statistical analysis on the endocrine data because they had certain characteristics of being pre menopausal. Falls were observed for LH & FSH. It woud appear that the drug does have agonist effects on gonadotrophins. There was no difference between the high and low doses in respect to the agonist effects but the numbers were small. Response data is available on 12 patients.

There has been one definite response. A phase II trial is now in progress.

### 1.2 The *in vitro* drug sensitivity of 8-chloro-cAMP in leukaemia and lymphoma

A.G. Bosanquet[1], A.R. Burlton[1], P.B. Bell[1], D. Talbot[2] & A.L. Harris[2]

*[1]Bath Cancer Research Unit, Royal United Hospital, Bath BA1 3NG; and [2]ICRF Clinical Oncology Unit, Churchill Hospital, Oxford OX3 7LJ, UK.*

8-Chloro-cAMP (8-Cl-cAMP) is a cAMP analogue with a novel antitumour action [Cho-Chung, Y.S. *et al.* 1991, *Pharmac. Ther.*, **50,** 1]. Whilst it is being investigated in a Phase I clinical trial in solid tumours, we have tested its *in vitro* drug sensitivity in leukaemia and lymphoma using the Differential Staining Cytotoxicity (DiSC) assay [Bosanquet, A.G. (1991, *Lancet*, **337,** 711]. Fresh isolated human tumour cells were incubated with 1.5–380 µg/ml 8-Cl-cAMP for the 4 days of the assay to identify an $LC_{90}$. Two of the three acute myeloid leukaemia (AML) specimens were sensitive *in vitro* ($LC_{90}$s = 1.5, 12 µg/ml) compared with the median of all specimens tested of 38 µg/ml. This confirms observations that AML cell lines are sensitive to this drug [Cho-Chung]. Non-Hodgkin's lymphoma and chronic lymphocytic leukaemia specimens showed less sensitivity with $LC_{90}$s of 24–>380 µg/ml. Two specimens that contained only normal cells at the end of the incubation period were very resistant to the drug ($LC_{90}$s≫ 380 µg/ml) suggesting a good *in vitro* therapeutic index. We correlated these results with the $LC_{90}$s of the similar drugs fludarabine (2-fluoro-ara-AMP, F1) and cladribine (2-Cl-deoxyadenosine, CDA). Whilst F1 and CDA were strongly cross resistant ($n = 30$, $r^2 = 0.72$, $P = 5 \times 10^{-9}$) there was little cross resistance of these drugs to 8-Cl-cAMP (8-Cl-cAMP *vs.* F1, $n = 30$, $r^2 = 0.07$, $P = 0.16$; 8-Cl-cAMP *vs.* CDA, $n = 31$, $r^2 = 0.19$, $P = 0.015$). These results suggest a role for 8-Cl-cAMP in leukaemias and lymphoma and especially against AML.

### 1.3 Antitumour activity *in vitro* of an inhibitor of Golgi assembly, brefeldin A

J.A. Plumb, A. Wise, M. O'Prey[1], L. Coggins[1] & P. Workman

*CRC Department of Medical Oncology, University of Glasgow and [1]Beatson Institute, Garscube Estate, Glasgow G61 1BD, UK.*

Brefeldin A, a fatty acid derivative of fungal origin, inhibits movement of membrane proteins from the endoplasmic reticulum to the Golgi and hence causes disassembly of the Golgi apparatus (Lippincott-Schwartz *et al.*, *Cell*, **60,** 821, 1990). We have investigated the potential antitumour activity of brefeldin A *in vitro* in a number of human tumour cell lines. The Golgi complex was visualised by the use of confocal microscopy and a fluorescent lipid probe, BODIPY ceramide, which specifically associates with Golgi membranes in living cells. Cytotoxicity ($ID_{50}$ 0.7–5.3 µM) was observed after exposure to Brefeldin A for only 1 h. Furthermore, the $ID_{50}$ concentration was close that required for disassembly of the Golgi apparatus. PtK1 cells, derived from the kangaroo rat kidney, are known to be resistant to the effects of brefeldin A on the Golgi apparatus (Ktistakis *et al.*, *J. Cell Biol.*, **113,**

1009, 1991). We have shown that these cells are also resistant to the cytotoxic effects of Brefeldin A such that no cell kill was observed at the limit of solubility of Brefeldin A (100 μg/ml, 357 μM).

It has been suggested that accumulation of cytotoxic drugs in intracellular compartments and increased vesicular trafficking may play a role in drug resistance. Treatment of multidrug-resistant breast (MCF7Adr) and ovarian (2780AD) cell lines with brefeldin A at concentrations shown to disrupt the Golgi apparatus had no effect on sensitivity to doxorubicin. This suggests that this is not an important mechanism in these cell lines even though we were able to demonstrate co-localisation of doxorubicin and BODIPY ceramide in the breast cell line.

These results suggest that brefeldin A has potent anti-tumour activity and that the mechanism of action may be related to effects on intracellular membrane trafficking.

### 1.4 Effect of essential fatty acids (N-6) on the growth of human colonic cancer cells

G.S. Lindsay & H.M. Wallace

*Departments of Medicine and Therapeutics and Biomedical Sciences, University of Aberdeen AB9 2ZD, UK.*

It has been suggested that the essential fatty acids (EFAs) of the n-6 series promote the growth of tumour cells while the EFAs of the n-3 series inhibit the growth of these cells. However there is some controversy as to whether all members of the n-6 series promote the growth of tumour cells. The aim of this study was to determine the effects of two EFAs, linoleic (C18:2 n-6; LA) and γ-linolenic (C18:3 n-6; GLA) acids, on the growth of human colonic carcinoma cells (HT115) in culture. Cells were grown in monolayer culture under standard conditions in medium supplemented with 10% (v/v) horse serum. Cells were exposed to the EFAs for 96 h and cell growth analysed by changes in cell number, protein content and DNA synthesis. Polyamine content was also measured as an index of the rate of cell growth. LA increased both protein content and cell number at concentrations of 5 and 20 g/ml with little effect on the cell viability. In contrast, GLA at the same concentrations had little effect on protein content by inhibited cell division, as measured by change in cell number, and viability (Table 1).

**Table I** Effect of LA and GLA on HT115 cell growth (*P < 0.05)

| *Treatment* | *Protein content (mg/plate)* | | *Cell number ( × 10⁻⁶)* | | *Viability (%)* | |
|---|---|---|---|---|---|---|
| | *LA* | *GLA* | *LA* | *GLA* | *LA* | *GLA* |
| Ethanol | 0.63 | 0.64 | 1.63 | 1.20 | 86 | 81 |
| 5 g/ml | 0.81* | 0.58 | 1.69 | 0.52 | 84 | 72 |
| 20 g/ml | 0.89* | 0.53* | 2.01 | 0.46 | 83 | 68 |

LA had little effect on polyamine content while GLA decreased spermine. Effects on DNA synthesis paralleled those on cell number. Clearly only LA has a stimulatory effect on the growth of these tumour cells while GLA is inhibitory as are the EFAs of the n-3 series.

### 1.5 Induction of apoptosis by withdrawal of serum in a p185*neu* transformed human mammary epithelial cell line

R.A. Harris[1], M. Page[1] & M.J. O'Hare[2]

*[1]Wellcome Research Laboratories, Beckenham, Kent BR3 3BS; and [2]ICR, Haddow Laboratories, Sutton SH2 5NS, UK.*

Apoptosis is a form of programmed cell death characterised by morphological alterations, which include chromatin condensation, blebbing of the plasma and nuclear membranes, the shrinkage of cell volume, and is often associated with the cleavage of DNA into nucleosomal unit lengths. It has been suggested that in developing malignancies, the rate of tumour growth is linked to the relative rates of cell proliferation and apoptosis. Many anti-tumour agents have been reported to act by inducing apoptosis, both *in vivo* and *in vitro*.

A conditionally immortalised human mammary luminal epithelial cell line, HB4A, derived by transfection of the $t_s$A58.U19 large T-antigen, was transformed by introduction of the mutant *neu* gene. After serum-withdrawal the parental HB4A line growth arrested, whereas the *neu* transformed derivative, N4.1, displayed all the characteristics of apoptosis. The alterations of cellular morphology produced by the phenomenon were observed during time-lapse photography, while the condensation of chromatin was confirmed by both DNA histogram analyses and electron microscopy. The nucleosomal cleavage of DNA is being examined by agarose gel electrophoresis. Apoptosis in N4.1 was abrogated by the addition of glucocorticoids, but not by other steroids.

These observations provide a model for the study of apoptosis in human mammary luminal epithelial cells. If apoptosis is a regulated pathway in human breast tumour cells, it could be manipulated for potential therapeutic use. Thus, it may ultimately be possible to induce apoptosis specifically in tumour cells.

### 1.6 Prediction of accumulation of $^{131}$I-meta-iodobenzylguanidine in neuroblastoma cell lines by means of reverse transcription and polymerase chain reaction

R.J. Mairs[1], A. Livingstone[1] & M.N. Gaze[2]

*[1]Department of Radiation Oncology, University of Glasgow, Garscube Estate, Glasgow G61 1BD, [2]The Meyerstein Institute of Clinical Oncology, The Middlesex Hospital, Mortimer Street, London W1N 8AA, UK.*

$^{131}$I-meta-iodobenzylguanidine (mIBG) is concentrated in sympathetic neurones, adrenal medullary cells and neural crest-derived tumours by an active process which is mediated by the noradrenaline transporter. Targeted radiotherapy of neuroblastoma using $^{131}$I-mIBG currently provides one of the best examples of selective anti-cancer therapy using radionuclides.

In order to identify those patients for whom $^{131}$I-mIBG therapy will be appropriate, and to estimate dosimetry and response to this form of treatment, a pre-therapy $^{131}$I-mIBG tracer study is usually carried out. This procedure, however, is only useful in patients with residual macroscopic tumour.

In the pursuance of a cost-effective alternative to time-consuming mIBG scintigraphic procedures of dubious precision and of a procedure which should be applicable to all patients irrespective of disease status, we evaluated the potential of reverse transcription followed by the polymerase chain reaction (RT-PCR) to predict mIBG uptake. The expression of the noradrenaline transporter gene was compared with that of the 28S rRNA gene in six human neuroblastoma cell lines and in three non-neural crest-derived cell lines. Transcription of the noradrenaline transporter gene was observed in five out of six neuroblastoma cell lines but in none of the control cells.

The RT-PCR procedure was specific (target mRNA sequences were absent in control cells, and the PCR product and its restriction endonuclease-derived fragments were of the predicted sizes), reproducible (coefficient of variation > 13.2%) and sensitive (detection limit < 50 ng of total RNA). A highly significant correlation was established (P < 0.01) between gene expression and active cellular accumulation of mIGB. It is suggested that semi-quantitative evaluation of noradrenaline transporter gene transcripts may be predictive of mIBG uptake by tumours *in vivo*.

### 1.7 Formation of DNA triplexes between oligonucleotides and N-myc

J.A. O'Donoghue[1], R.J. Mairs[1] & T. Brown[1]

[1]*Department of Radiation Oncology, University of Glasgow and* [2]*Department of Chemistry, University of Edinburgh, UK.*

Amplification of N-myc has been reported in several tumour types including neuroblastoma, where it is associated with a poor clinical prognosis. It may therefore be a good candidate for targeted radionuclide therapy using triplex forming oligonucleotides as targeting vectors. Oligonucleotides were designed to form DNA triplexes with a 25 bp homopurine-homopyrimidine region at the 5′ end of the N-myc gene as shown in the table.

**Table** Sequences of the target duplex and oligonucleotides

| | |
|---|---|
| N-myc Target Duplex | |
| 5′-GGTAAAGCCGC TTTCCTCTCCTTTCTCCCTCCCCCT TGTC TGCGCCACA-3′ | |
| 3′- TTTCGGCG AAAGGAGAGGAAAGAGGGAGGGGGA ACAGA CGCGGTGTCGG-5′ | |
| Targeting Oligonucleotides | |
| | Nt1 5′-AGGGGGAGGGAGAAAGGAGAGGAAA-3′ |
| | Nt2 5′-AAAGGAGAGGAAAGAGGGAGGGGGA-3′ |
| | Nt3 5′-TGGGGGTGGGTGTTTGGTGTGGTTT-3′ |
| | Nt4 5′-TTTGGTGTGGTTTGTGGGTGGGGGT-3′ |

The boxed regions indicate the anticipated sites of triplex formation.

In an assay of triplex formation 5′-end labelled oligonucleotides were incubated with varying concentrations of the N-myc target duplex in 20 mM Tris-Cl (pH 7.2) 10 mM $MgCl_2$ 10% sucrose. For the triplex inhibition assay the incubation buffer was 20 mM Tris-Cl (pH 7.2) 2 mM EDTA 10% sucrose. Polyacrylamide gel electrophoresis was performed for 3 h at 500 V at room temperature.

Subsequently, 3′-end labelled N-myc target duplex was incubated with varying concentrations of the targeting oligonucleotides. Electrophoresis was performed for triplex forming and triplex inhibition assays as above. We now have data consistent with DNA triplex formation between the targeting oligonucleotides Nt2 and Nt4 and the N-myc target duplex. Neither of the other two N-myc targeting oligos Nt1 and Nt3 forms triplexes. The formation of triplexes between N-myc and Nt2 or Nt4 is inhibitable by incubation and electrophoresis in the presence of 2 mM EDTA.

Preliminary data suggest that Nt4 forms a stronger association than Nt2 with N-myc.

### 2.1 Oncogene status of bladder tumours: the value of multiparametric flow cytometry

B.K. Shenton, K. Mellon, S. Wilkinson, I. Brotherick, B. Angus, C.W.V. Horne, T.W. Lennard & D.E. Neal

*Department of Surgery, Medical School, University of Newcastle, Newcastle NE2 4HH, UK.*

Mutations and over-expression of the p53 gene have been reported to be one of the most common genetic alterations in human malignancy. The determination of such abnormalities is usually by immunoprecipitation, sequencing or by immunocytochemistry. In the present study we have examined tumour cells from frozen bladder tumours of 14 patients using flow cytometry. The panel of FITC conjugated antibodies used included four to different p53 epitopes, and one to c-erbB2. We employed a permeabilisation process to the cells using saponin which allowed the simultaneous assessment of surface and intercellular components. Titration of the conjugated antibodies was performed to determine their optimum concentrations.

The presence of aneuploidy was highly associated with p53-240 ($P = 0.007$), p53-DO7 ($P = 0.035$) and c-erbB-2 ($P = 0.0023$) but not with expression of p53-CM1 or p53-1801. Similar results were seen in 12 tumour lines tested where the tumours could be separated into different groups based upon their p53 epitope expression. High correlations were also found between the presence of c-erbB2 and both p53-DO7 ($P = 0.035$) and p-53-240 ($P = 0.008$) expression on the tumour cells.

These studies indicate that multiparameter flow cytometry may be a valuable investigative tool in the analysis of tumour cells where both nuclear DNA and the expression of oncogene products may be determined. It also suggests that in order to determine p53 expression a number of antibodies may be useful.

### 2.2 Somatic allelic losses at DCC, APC, p53 and NM23-H1 tumour suppressor gene (TSG) loci in translational cell carcinoma of the bladder (TCC)

S.F. Brewster[1,2], K.W. Brown[2] & J.C. Gingell[1]

[1]*Department of Urology, Southmead Hospital, Bristol BS10 5NB; and* [2]*Department of Pathology, School of Medical Sciences, Bristol BS8 1TD, UK.*

Thirty-one human TCCs were examined for loss of heterozygosity (LOH) at four TSG loci and chromosome 11p15 using the following DNA probes in a restriction fragment polymorphism analysis: DCC [*d*eleted in *c*olorectal *c*arcinoma] (18q21.3), p15-65, SAM 1.1, JOSH 4.4, DCC 1.9 – B. Vogelstein, John Hopkins; p53 (17p13.1), pMCT35.1; nm23-H1 (17q21.3, pNM23-H1 – P. Steeg, NIH); APC [*a*denomatous *p*olyposis *c*oli] (5q21), FB54D and p2.1 VNTR (11p15). Results are expressed as number showing LOH/number informative heterozygous samples.

| *Gene* | *DCC* | *p53* | *nm23-H1* | *APC* | *11p15* |
|---|---|---|---|---|---|
| Stage | | | | | |
| pTa | 3/14 | 1/5 | 0/13 | 2/9 | 2/8 |
| pT1 | 1/5 | 1/2 | 0/4 | 0/4 | 1/4 |
| pT2/3 | 4/5 | 1/4 | 2/7 | 0/1 | 3/7 |
| Total | 8/24 | 3/11 | 2/24 | 2/14 | 6/19 |
| Grade | | | | | |
| 1 | 0/3 | 1/1 | 0/1 | 0/1 | 0/1 |
| 2 | 5/15 | 0/5 | 1/16 | 2/10 | 4/11 |
| 3 | 3/6 | 2/5 | 1/7 | 0/3 | 2/7 |
| Total | 8/24 | 3/11 | 2/24 | 2/14 | 6/19 |

LOH at p53, nm23-H1, APC and 11p15 occurred in 27%, 8%, 14% and 32% tumors respectively, those at nm23-H1 seen only in high stage TCCs.

LOH at DCC, observed in 33% TCCs, occurred proportional to increasing tumor grade and stage. Five informative grade 2 TCCs were recurrent, of which three showed LOH at DCC; two of the three pTa DCC-deleted tumors were recurrent. In addition, four RFLP markers located on 18q both proximal and distal to DCC (pL2.7, OLV11 A8, pOS-4 and pL159-I) failed to detect LOH in TCCs heterozygous at DCC, suggesting the DCC TSG is the target for deletion in invasive and recurrent bladder cancer (supported by a SWRHA research grant).

### 2.3 Loss of heterozygosity on chromosome 3 and 17 in head and neck squamous cell carcinomas indicates new regions with allelic imbalance

R.E. Adamson[1], C. Tsiriyotis[1], A.S. Jones[2] & J.K. Fields[1]

*Department of* [1]*Clinical Dental Sciences and* [2]*Otorhinolaryngology, University of Liverpool, PO Box 147, Liverpool L69 3BX, UK.*

Loss of heterozygosity (LOH) has been successfully used to identify novel regions that may contain tumour suppressor genes. We have investigated LOH on chromosomes 3 and 17 in squamous cell carcinoma of the head and neck (SCCHN) using panels of microsatellite markers. DNA was isolated from 38 SCCHN and their respective normal tissues. All tumour specimens used in this study contained more that 50% tumour tissue compared with normal. PCR reactions were performed in a 50 μl reaction volume and contained 200 ng of genomic DNA, 500 μM dNTP, 10 pmoles of each forward and reverse microsatellite primers and 0.5 units of Taq DNA polymerase. The DNA was amplified for 25 cycles of 94°C for 30 s at the appropriate primer annealing temperature for 30 s and 72°C for 1 min. 10 μl of the amplified PCR reaction mixture was electrophoresed overnight on a 10% acrylamide gel. The acrylamide gels were silver-stained.

LOH on chromosome 3 was determined with 12 microsatellites and 46% (17/37) of the tumours analysed had LOH at one locus. The highest incidence of LOH was found at D3S1293 (33%) at 3p24. A clear association was found between LOH on chromosome 3 and a poor clinical prognosis, as judged by level of differentiation, nodes at pathology and TNM staging. LOH was also analysed on chromosome 17 with nine microsatellite markers. LOH on 17p in SCCHN was found in 47% (18/38) and often involved TP53 at 17p13.1 (42%) but more noticeably involved the CHRNB1 locus at 17p12-p11.1 (56%). Fifteen tumours also showed LOH on 17q.

These results indicate that two regions, with allelic imbalance at 3p24 and 17p12-p11.1 have been found in SCCHN and these may represent sites with novel tumour suppressor genes.

### 2.4 Deletions from chromosome 17q in ovarian cancer

I. Hickey[1], M.J. Murphy[2], D.P. Harkin[1], D.W. Bell[2], S. McIlroy[3], A.N. Cranston[1], R.J. Atkinson[2], M. Humphries[3], J. Devlin[1] & S.E.H. Russell[3]

*[1]School of Biology and Biochemistry, [2]Department of Oncology and [3]Department of Medical Genetics, The Queen's University of Belfast.*

The involvement of chromosome 17q in sporadic epithelial ovarian cancer has been demonstrated by a number of groups on the basis of loss of heterozygosity (LOH) studies. Linkage studies in families with early onset breast-ovarian cancer have indicated a gene (BRCA1) mapping to 17q12-21 which confers an inherited susceptibility to breast and ovarian cancer.

We have examined 70 sporadic epithelial ovarian tumours and matched control DNA with 20 markers from chromosome 17 (two from the short arm and 18 from the long arm).

The highest LOH was found towards the telomere with markers in the region 17q25.

Approximately 60% of tumours displayed a pattern of LOH consistent with loss of an entire chromosome 17. Analysis of nine tumours with partial 17q deletions suggests a common region of deletion which maps between the markers THH59 and RMU3.

Translocations involving chromsome 17 were observed in a number of cell lines derived from epithelial ovarian tumours.

### 2.5 Molecular cytogenetic analysis of the topoIIα locus

W.N. Keith, F. Douglas & J. Coutts

*Department of Medical Oncology, CRC Beatson Laboratories, Garscube Estate, Switchback Road, Bearsden, Glasgow G61 1BD, UK.*

The levels of topoIIα within a cell will in part determine its sensitivity to the cytotoxic effects of topoisomerase inhibitory drugs. The topoIIα locus is situated on chromosome 17q in a region frequently altered during the development of many tumour types. The ERBB2 oncogene which is on chromosome 17q, is frequently amplified in breast cancer. We have shown by Southern blot analysis that in three out of six tumours with ERBB2 amplification that the amplicon can encompass ERBB2, RARα and topoIIα. Interestingly, although these three genes are co-amplified the levels of amplification of each locus within each tumour can vary. In tumour 2, all three genes are amplified to a similar extent, whereas tumours 1 and 3 display higher levels of topoIIα and RARα amplification respectively. These data suggest amplicon evolution to be a complex process. The amplification of topoIIα would be expected to sensitise the tumour to topo inhibitory drugs should topoIIα be expressed. We have shown by both Western blot analysis and biochemical assay that amplification of topoIIα in breast cancers can result in high expression. In order to study genetic change at the topoIIα locus further we have used fluorescence *in situ* hybridisation (FISH). We have analysed a number of cell lines for topoIIα copy number by FISH. The CALU3 cell line is a good model for the amplification observed in breast cancer as it has co-amplified ERBB2, RARα and topoIIα. By FISH, CALU3 has five copies of topoIIα whereas the cell lines L-DAN and SKMES have three and two copies respectively. However, CALU3 expresses 10-15 times more topoIIα than the other cell lines suggesting the possibility that gene amplification may disrupt normal control of gene expression. FISH is an ideal method for studying genetic change as it can accurately determine gene copy number and heterogeneity within a cell population. We are now applying FISH to clinical samples to examine genetic change on chromosome 17 and its histological distribution.

### 2.6 p53 gene mutation predicts response of colorectal cancer to chemotherapy

M. Pickard, M. Brett, B. Green, A. Howel-Evans, G. Poston & A. Kinsella

*Department of Surgery, Liverpool University, Liverpool, UK.*

Mutation of the p53 gene is the most common cancer related genetic change known at the gene level and occurs in 70% of colon carcinomas. Wild type p53 produces a nuclear phosphoprotein, believed to be involved in cell cycle regulation, and has a very short half life. Mutant forms of p53 have a longer half life and are therefore detectable using immunohistochemistry. The ABC method of immunohistochemical staining was used on paraffin sections of the primary tumours to investigate whether p53 gene mutation can be used to predict response to chemotherapy. Two independent observers scored the sections positive or negative for the presence or absence of p53 protein, respectively.

Thirty-three colon carcinoma patients were tested who had each received 5-FU (370 mg/m$^2$) and Folinic acid (200 mg/m$^2$) for five consecutive days every 28 days for up to 6 months of treatment. CAT scans of an index lesion after 3 and 6 months of treatment showed either response (>50% regression), static disease or progressive disease. Results were as follows:

| | *Mutant p53* | *Wild type p53* |
|---|---|---|
| Response/Static disease | 4 | 12 |
| Progressive disease | 12 | 5 |

Mutant types were significantly less likely to respond to chemotherapy than wild type (P = 0.011, Fisher's exact test) and response was unrelated to age, sex or tumour grade. Assessment of p53 status may assist in patient selection for bimodulated 5-FU chemotherapy.

**2.7 Over-expression and amplification of the MDR1 gene in the doxorubicin resistant human bladder cancer cell line, KK47/ADM**

S.C. Clifford[1], S. Naito[3], D.E. Neal[2] & J. Lunec[1]

[1]*Cancer Research Unit and* [2]*Department of Surgery, Medical School, University of Newcastle upon Tyne, UK; and* [3]*Department of Urology, Kyushu University, Fukoka, Japan.*

Establishment of the drug resistant human bladder cancer cell line, KK47/ADM has recently been described. Following stepwise selection in increasing concentrations of doxorubicin, KK47/ADM was 271 times more resistant to doxorubicin than its KK47 parent. Preliminary characterisation revealed KK47/ADM to have a classical multidrug resistance phenotype. Elevated levels of P-glycoprotein were observed together with decreased drug accumulation caused by an increased active efflux, which was reversed in the presence of verapamil. Cross resistance to the anthracyclines, vinca alkaloids and etoposide (but not cisplatin or methotrexate) was observed (Kimiya *et al*., *J. Urol*., **148,** 441–445, 1992).

mRNA levels of the MDR1 gene (which encodes P-glycoprotein) were determined by a quantitative PCR-based transcript assay and Northern blot hybridisation. While the sensitive parental line, KK47, had low levels similar to those seen in primary superficial bladder tumors, from which it was derived, the KK47/ADM resistant line had 55-fold higher levels ($P = 0.0023$), more comparable with the levels found in high MDR1 expressing adrenal tissue and 10-fold higher than any bladder tumor sample we have analysed. Southern blot analysis of MDR1 gene copy numbers revealed a 5-fold increase in the gene copy number of the KK47/ADM cell-line when compared to that of its parental KK47 line. Results thus show increased transcription to be the predominant mechanism of MDR1 overexpression in the KK47/ADM cell line.

Verapamil could not completely overcome the resistance of KK47/ADM to doxorubicin (Kimiya *et al.*) suggesting that while overexpression of the MDR1 gene is the principal mechanism of drug resistance, other mechanisms may be present which play a smaller but none the less contributory role. Transcript levels of the MRP gene (associated with non-P-glycoprotein mediated MDR) were determined and found to be 1.7-fold higher in KK47/ADM than its parental line, though this result was not significant ($P = 0.26$).

**2.8 N-myc gene copy number in neuroblastoma cell lines and resistance to experimental treatment**

A. Livingstone[1], R.J. Mairs[1], J. Russell[1], J. O'Donoghue[1], M.N. Gaze[2] & T.E. Wheldon[1]

[1]*Department of Radiation Oncology, University of Glasgow, Garscube Estate, Glasgow G61 1BD;* [2]*The Meyerstein Institute of Clinical Oncology, The Middlesex Hospital, Mortimer Street, London W1N 8AA, UK.*

The N-myc oncogene is amplified in approximately 30% of neuroblastoma. It is well established that cases of neuroblastoma with amplified N-myc have markedly poorer prognosis than those in which N-myc copy number is not elevated. The mechanism for this association is not known but may be related to cellular resistance to radiation or cytotoxic drugs.

Seven human neuroblastoma cell lines were used to investigate the relationship between N-myc copy number or expression and sensitivity to ionising radiation and to cisplatin.

N-myc copy number was assessed by Southern blotting and hybridisation using the p-Nb1 probe. The signal produced by DNA from the cell lines was compared with that of single copy N-myc from normal human placental DNA. A range of N-myc copy numbers from 1–800 was found. Expression levels of N-myc m-RNA were compared by 'dot blotting' and subsequent hybridisation to the p-Nb1 probe. Radiosensitivity was assessed by surviving fraction at 2 Gy ($SF_2$) following $^{60}Co$ gamma irradiation. Values ranged from 0.13–0.52. Sensitivity to cisplatin was indicated by comparison of iso-effective concentrations (concentration required to produce 1 log cell kill). These ranged from 7.5–13 μM.

Cisplatin studies showed a correlation between N-myc copy number (though not expression) and resistance to this drug. If this relationship is causal it may explain why treatment fails in those patients with elevated N-myc copy number. However, no correlation was found between N-myc copy number or expression and sensitivity to radiation. It is possible that N-myc amplification confers resistance to some but not all treatments used in the therapy of neuroblastoma. Further investigations along these lines may lead to the investigation of agents which are most appropriate for the treatment of neuroblastoma with amplified N-myc gene.

**3.1 The activity of deoxyspergualin in multidrug resistant cells**

J.A. Holmes & P.R. Twentyman

*MRC Clinical Oncology & Radiotherapeutics Unit, Hills Road, Cambridge CB2 2QH, UK.*

15-deoxyspergualin (DSG) is a synthetic analogue of the immunosuppressive antitumour antibiotic spergualin that was first isolated from culture filtrates of *Bacillus laterosporus* (Takeuchii *et al*., *J. Antibiot*., **34,** 1619, 1981). The compound has been shown to possess potent *in vitro* and *in vivo* antitumour activity and is currently in the NCI decision network. DSG has also been shown to bind to the human constitutive heat shock protein Hsp70. Cyclophilin (the cyclosporin A (CsA) binding protein), FK506 binding protein, and heat shock proteins are all involved in the regulation of protein folding, which suggests that a common mechanism of action may exist for these immunosuppressants (Nadler *et al*., *Science*, **258,** 484, 1992). As CsA and FK506 are natural products possessing potent immunosuppressive properties and also the ability to act as modifiers of classical multidrug resistance (MDR), we decided to examine the activity of DSG in MDR cells. DSG contains the polyamine spermidine within its structure and can therefore be considered a spermidine analogue. Bovine serum (BS) amine oxidase catalyses the oxidative deamination of spermidine to produce aminoaldehyde, ammonia and $H_2O_2$. It is thought that these aminoaldehydes are responsible for the toxicity of polyamines *in vitro* in the presence of BS (Kunimoto *et al*., *J. Antibiot*., **38,** 899, 1985). For this reason all experiments involving incubation of cells in medium were performed in duplicate using both BS and horse serum (HS), which is low in amine oxidase content. We used the mouse tumour cell line EMT6/P and the human small cell lung cancer line H69/P together with their P-glycoprotein (Pgp) hyperexpressing sublines EMT6/AR1.0 and H69/LX4. Cytotoxic effects were determined using the MTT colorimetric assay with a 3 day (EMT6) or 6 day (H69) assay duration. Mean IC50 values in BS were 1.6, 2.8, 11, 15 μg/ml for EMT6/P, EMT6/AR1.0, H69/P and H69/LX4. Corresponding values in HS were 6, 8, 22, 40 μg/ml respectively. The lines are therefore 2–4 fold more sensitive to DSG in BS than in HS. However, the MDR sublines showed only minimal cross-resistance to DSG. At 5–100 μg/ml, DSG did not enhance the accumulation of [$^3$H]daunorubicin in EMT6/AR1.0. Furthermore, DSG (0.5–10 μg/ml) did not alter the $IC_{50}$ of doxorubicin in H69/LX4 cells. Pgp in membranes from H69/LX4 cells was photoaffinity-labelled with [$^3$H]azidopine. DSG did not inhibit this labelling. Although therefore DSG appears to exert its immunosuppressive actions via a similar mechanism to that of CsA and FK506, our results show that it does not share their ability to modify Pgp-mediated MDR. They also confirm that the cytotoxicity of DSG is increased in the presence of BS compared to HS.

### 3.2 Competitive inhibition by genistein of the ATP-dependent daunorubicin transport in an *MRP* overexpressing, MDR lung cancer cell line

C.H.M. Versantvoort, H.J. Broxterman, J. Lankelma, N. Feller & H.M. Pinedo

*Department of Medical Oncology, Free University Hospital, Amsterdam, The Netherlands.*

In several multidrug resistant tumor cell lines without overexpression of P-glycoprotein (non-Pgp MDR), a decreased accumulation of drugs has been shown to contribute to the resistance. The daunorubicin accumulation in the GLC4/ADR non-Pgp MDR lung cancer cells was shown to be decreased due to an enhanced energy-dependent DNR efflux. The GLC4/ADR cells do overexpress the *MRP* gene[1,2], which was recently discovered to be overexpressed in another non-Pgp MDR lung cancer cell line. We now further characterised the daunorubicin transport and its dependence on cellular ATP levels. Furthermore, the effects of the isoflavonoid genistein on the decreased daunorubicin accumulation in the GLC4/ADR cells were studied.

As measured by a relative increase in steady-state accumulation of daunorubicin, the active efflux of daunorubicin out of the GLC4/ADR cells appeared to be a saturable process with an apparent $K_M$ value of DNR of 1.4 μM. Genistein reversed the decreased daunorubicin accumulation in the GLC4/ADR and several other non-Pgp MDR cell lines. In contrast, the daunorubicin accumulation in Pgp MDR cell lines was not increased by 200 μM genistein. Furthermore, the apparent $K_M$ value of daunorubicin in the GLC4/ADR cells was increased by genistein, suggesting that this agent is a competitive inhibitor of the active DNR transport.

Marked inhibition of the daunorubicin transport activity was found at cellular ATP concentrations below 2 mM. Thus the daunorubicin transport activity in intact GLC4/ADR cells is already impaired by a rather modest cellular ATP depletion. This might open ways to enhance the toxic effects of drugs in MDR cells.

1. Cole, S.P.C. & others. (1992). *Science*, **258**, 1650–1654.
2. Zaman, G.J.R. & others. (1993). *Cancer Res.*, **53**, 1747–1750.

### 3.3 Influence of temperature, pH and the chemical modifier, genistein, on intracellular anthracycline distribution in non-Pgp MDR cell line, COR-L23/R

T. Rhodes & P.R. Twentyman

*MCR Clinical Oncology & Radiotherapeutics Unit, Hills Road, Cambridge CB2 2QH, UK.*

COR-L23/R is a human MDR cell line which does not overexpress the MDR-1 gene or its product, P-glycoprotein (Pgp), but has high levels of the alternative transporter molecule, MRP (Cole *et al. Science*, **258**, 1650, 1992). Following acute exposure to doxorubicin (DOX), the total cellular drug accumulation and intracellular drug distribution are strikingly different in COR-L23/R compared with the parental line COR-L23/P. It is known that cellular accumulation of DOX is pH and temperature dependent and that extracellular pH in tumours is frequently 0.5 pH units lower than in normal tissues. We have therefore compared the effects of changing temperature and/or extracellular pH on the anthracycline distribution in COR-L23/P and COR-L23/R cells. Cells grown on glass coverslips were treated with DOX (20 μM, 1 h), rinsed in ice-cold PBS, inverted onto a slide and visualised using the Biorad MRC-600 laser-assisted confocal fluorescence microscope. In COR-L23/P at 37°C and pH 7.4, fluorescence was predominantly nuclear with only sparse, punctate cytoplasmic fluorescence. By contrast, fluorescence in COR-L23/R was mainly confined to groups of perinuclear cytoplasmic vesicles with little nuclear fluorescence. In COR-L23/P, changing pH at 37°C from 5.8 to 8.6 resulted in increased nuclear fluorescence without major changes in distribution. Marked alterations were however observed in distribution with changing extracellular pH in the resistant line. At 37°C and pH 5.8, fluorescence was weak and wholly restricted to the cytoplasm, whereas at pH 8.6 it was stronger, mainly nuclear and only weak cytoplasmic fluorescence was observed. At a temperature of 22°C and pH 8.6 the fluorescence distribution in COR-L23/R became identical to that in COR-L23/P. This result could not be achieved by low temperature alone. A reduction in temperature to 4°C (at pH 7.4) resulted in low fluorescence, confined to the cytoplasm, in both the resistant and parental cells. It appears therefore that drug accumulation and distribution in COR-L23/R cells are dependent upon metabolic rate and upon extracellular pH, possibly due to pH-dependent changes in DOX ionisation.

A number of chemical agents have been suggested as modifiers of non-Pgp MDR and an agent of current interest is the tyrosine kinase inhibitor genistein, which has been found to alter intracellular DOX distribution (Takeda *et al.*, *Proc. AACR*, **33**, 476, 1992), and daunorubicin (DNR) accumulation (Versanvoort *et al.*, *Br. J. Cancer*, **Dec**, 1993) in other non-Pgp MDR cell lines but not classic MDR cell lines. In COR-L23/R a non-toxic dose of genistein (400 μM, 1 h) completely restored DNR accumulation to control levels, whilst having no effect on the parental line. Confocal studies observing the effects of genistein on the intracellular DNR distribution in COR-L23/R confirmed the increased levels of drug and also patterns of intracellular distribution similar to those in parental cells.

### 3.4 *In vitro* modification of resistance to MDR-related drugs in acute myeloid leukaemia (AML)

J. Sargent, A. Elgie, P. Alton & C. Taylor

*Haematology Research, Pembury Hospital, Pembury, Kent TN2 4QJ, UK.*

The multidrug resistance (MDR) phenotype has been shown to effect the uptake and accumulation of anthracyclines and etoposide, the drugs used in first line treatment of AML. Tested against cell lines, verapamil (Ver), cyclosporin A (CsA) and tamoxifen (Tam) have been shown to chemosensitize cells which are resistant to these drugs.

We describe a study to test the ability of these agents to modify *in vitro* resistance to doxorubicin (Dox), mitoxantrone (Mit) and etoposide (VP16) using fresh blast cells from 18 patients with AML, 13 on presentation and five after previous treatment. The MTT assay was used to measure cell viability after 48 h continuous drug exposure. Log dose response curves were constructed for each test and if >30% of cells survived at 1 μg/ml for the anthracyclines and 25 μg/ml for VP16 the cells were deemed resistant. The effect of the modifier was determined by comparing the area under the curve for drug + modifier with that of drug alone. One hundred and nineteen comparisons were made overall; 41 showed an increase in sensitivity on co-incubation with a modifier. CsA (4 μM) was the most effective sensitizer showing significantly increased cytotoxicity in 24/41 (59%) comparisons ($P < 0.03$). Indeed, blast cells from 7/8 patients resistant to Dox were rendered sensitive with the addition of CsA. Ver (3.3 μg/ml) increased sensitivity in 12/53 (23%) cases and Tam (10 μM) in 4/25 (16%) cases. The effect of individual modifiers was variable between patients ranging from 7/9 tests in one patient to only 1/9 tests in another. There was no difference overall in the number of cases of chemosensitization in the group of patients who had previously received treatment.

The expression of the MDR product P-glycoprotein (Pgp) was assessed using immunocytochemistry on cytospin preparations of the cell suspension used in the MTT assay and the monoclonal antibodies C219, JSB-1 and MRK16. Preliminary data suggest no correlation between Pgp expression and chemosensitization. More sensitive techniques may be required to establish Pgp expression in the cells exhibiting resistance modification.

These data suggest CsA may be the best chemosensitization agent overall in AML. They also support the clinical use of drug modification regimes identified by *in vitro* screening techniques for individual patients.

### 3.5 Resistance to platinum-based drugs in ovarian carcinoma

J. Sargent, A. Elgie, C. Taylor, P. Alton & J.G. Hill

*Haematology Research, Pembury Hospital, Pembury, Kent TN2 4QJ, UK.*

Resistance to front line agents, such as cisplatin and its analogue carboplatin, remains a major problem in the treatment of ovarian cancer. This resistance, whether inherent or acquired, is thought to be due to increased intracellular detoxification.

We describe a study firstly, to identify resistance to these agents and secondly, to attempt to modify this resistance *in vitro*. One hundred and sixty-seven samples of ascitic fluid and 160 biopsy samples from 206 patients with ovarian adenocarcinoma were continuously exposed to four concentrations of cisplatin and carboplatin for 48 h. The cell viability was then assessed using the MTT assay. The success rate for the assay is currently 80%. Log dose response curves were calculated and if >30% of cells survived at 5 μg/ml cisplatin or 50 μg/ml carboplatin, the cells were deemed to be resistant. Cisplatin and carboplatin produced a similar cell kill in 76% of cases tested. The resistance rates for these agents in a group of untreated patients were 71% for cisplatin and 54% for carboplatin; the resistance rate for carboplatin increased to 65% in a group of patients who had received previous therapy. The assay results showed a positive correlation with the clinical outcome in 18 of 25 (60%) untreated patients ($P = 0.018$). Sixty percent of patients treated with a drug to which their tumour was sensitive showed a complete clinical response compared with only 10% of those whose tumour was resistant.

One of the mechanisms involved in platinum resistance is thought to be increased detoxification through the glutathione (GSH) pathway. We attempted to reduce intracellular levels of GSH *in vitro* using buthionine sulfoxamine (BSO; 100 μM) and inhibit glutathione-S-transferases (GSTs) with ethacrynic acid (ETH; 6.5 μM). Modification effect was assessed by comparing the area under the curve with that of drug alone. Forty-nine comparisons were made of the effect of these agents on cisplatin and carboplatin cytotoxicity using fresh cells from 13 patients. There was a marked variation between patients in the effect of co-incubating with BSO or ETH. BSO increased the sensitivity to carboplatin in three patients and ETH increased sensitivity to cisplatin in two patients. Indeed, in three patients, resistant cells were rendered sensitive by chemosensitization.

Variation between patients indicates the importance of *in vitro* screening for an effective modifier before treatment and these results support the clinical use of these resistance modifiers in the treatment of ovarian cancer.

### 3.6 Factors involved in cellular resistance to the bioreductive drug E09 and related mitosenes

I.J. Stratford[1], N. Robertson[1], A. Haigh[1], C. Moody[2], P. Workman[3] & G.E. Adams[1]

*[1]MRC Radiobiology Unit, Chilton, Didcot, Oxon OX11 ORD; [2]Department of Chemistry, University of Loughborough, Loughborough LE11 3TU; [3]Zeneca Pharmaceuticals, Mereside, Alderley Park, Macclesfield, Cheshire SK10 4TG, UK.*

Twenty-three human tumour cell lines (lung, breast and colon) have been evaluated for their sensitivity to the quinone based anti-cancer drug E09. Sensitivity has been compared with the intra-cellular levels of DT-diaphorase and cell lines showing the lowest enzyme activity are the most resistant to E09. Dicoumarol, an inhibitor of DT-diaphorase, renders cells containing high levels of enzyme resistant to E09. Further, using pairs of Chinese hamster cells with varying levels of DT-diaphorase, resistance is observed in those cells with low levels of reductase. Taken together these results are consistent with a dominant role for two-electron reduction, catalyzed by DT-diaphorase, in the bioactivation of E09.

Novel mitosenes related to E09 and mitomycin C have been synthesized. One of these (RB91008, an aziridinyl cyclopropamitosene) is 50 × more potent than E09 in V79 cells (3 h exposure in air). Treatment of cells with dicoumarol protects against the action of RB91008. Replacement of the aziridine in RB91008 with methoxy only has a slight effect on the ability of the compound to act as a substrate for DT-diaphorase but potency is reduced 1000 ×. These results illustrate the importance of reductive activation for controlling toxicity but also suggest that other processes can play a crucial role in controlling cellular resistance to these quinoid compounds.

### 3.7 Enhancement of bioreductive drug activity in experimental murine tumours by the nitric oxide synthase inhibitor nitro-L-arginine

G.E. Adams, P.J. Wood, J. Sansom, S. Butler & I.J. Stratford

*MCR Radiobiology Unit, Chilton, Didcot, Oxon OX11 ORD, UK.*

Nitric oxide is an endogenous vasodilator responsible for maintaining blood vessel potency, and is generated *in vivo* from L-arginine by nitric oxide synthase (NOS). NOS activity may be inhibited by agents such as nitro-L-arginine (NOARG) resulting in vasoconstriction. We have recently demonstrated using $^{31}$P mrs that the transplantable murine tumour SCCVII and various spontaneous murine mammary adenocarcinomas can respond to NOARG. This was observed as a change in phosphorous metabolism indicating increased tumour hypoxia.

The induction of tumour hypoxia is sufficient to activate bioreductive drugs, in particular the agent RB 6145 developed in this Laboratory. The drug is a substituted 2-nitroimidazole which, on reductive activation by cellular reductase, is converted to a powerful DNA cross-linking agent. It is highly active in various experimental solid tumours.

Treatment *in vivo* of the KHT murine tumour, by combination of nitro-L-arginine and RB 6145, produced a large anti-tumour effect when measured by a post-treatment excision cell survival assay. Single doses of RB 6145, in the range 100–300 mg/kg, were followed 15 minutes later by single doses of nitro-L-arginine 5–20 mg/kg. For the higher dose ranges, tumour cell-kill as measured by relative cell survival, exceeded five decades in the majority of tumours.

*In vivo* results including both tumour response and $^{31}$P mrs will be used to illustrate potential clinical value of this approach to overcome relatively inaccessible and chemo-resistant tumour cells.

### 3.8 Effect of salvage pathways on drug resistance

M. Pickard & A.R. Kinsella

*Department of Surgery, University of Liverpool, PO Box 147, Liverpool, L69 3BX, UK.*

Resistance of human tumour cells to chemotherapeutic agents has been a common clinical problem. The effect of nucleoside salvage in combination with the antimetabolites 5-fluorouracil, N-(phosphonacetyl)-L-aspartate and methotrexate was investigated. A series of cell lines of common genetic origin was used which exhibit a range of differing phenotypes from normal (KMS), through immortalized (KMST) to aggressively tumourigenic (KN-NM) (Namba *et al.*, 1987) (Kinsella *et al.*, 1990).

Kinsella and Haran (1991) demonstrated that the intrinsic sensitivities of methotrexate and PALA showed increasing resistance to parallel increasing tumourigenicity with these cell lines and this was unrelated to amplification of the respective target genes. Dipyridamole, a nucleoside transport inhibitor, was used in combination with the respective drugs in standard survival assays, in normal foetal bovine serum, to assess the contribution of the salvage pathways to resistance.

The KN-NM tumourigenic cell line was totally resistant to PALA up to a concentration of 1 mM compared to an $LD_{50}$ of 50 μM after the addition of dipyridamole. The KMS normal cell line had an $LD_{50}$ of 5 μM and 60 μM respectively in the presence and absence of dipyridamole. Methotrexate gave an $LD_{50}$ of 2.5 μM without dipyridamole and 0.7 μM with dipyridamole for the KN-NM cell line. This difference has been eradicated with the KMST cell line which gave an $LD_{50}$ of 0.4 μM both with and without dipyridamole. The KN-NM cell line had an $LD_{50}$ of 2.5 μM with 5-FU both with and without dipyridamole which fell to 0.9 μM 5-FU with and without dipyridamole for the KMS normal cell line. These data suggest that the salvage pathways are having a marked effect on resistance of the cell lines to PALA, only a moderate effect with methotrexate and no effect with 5-FU.

Keywords: Methotrexate; PALA; 5-Fluorouracil

### 3.9 Constitutive and induced heat shock protein levels in human cancer cell lines

E.H. Richards[1], J.R.W. Masters[1] & J.A. Hickman[2]

*[1]University College London, 3rd Floor, 67 Riding House St., London W1P 7PN; [2]CRC Molecular and Cellular Pharmacology Group, School of Biological Sciences, University of Manchester, Manchester M13 9PT, UK.*

Testicular germ cells are acutely sensitive to damage induced by chemotherapeutic drugs, irradiation and heat. This response to various forms of stress may explain why metastatic testis tumours, in contrast to most other types of advanced cancer, can be cured in over 80% of cases using cisplatin-based combination chemotherapy. To investigate the molecular basis of the stress response, we compared the sensitivities *in vitro* of human testis and bladder cancer cell lines to chemotherapeutic drugs and heat shock, and measured their constitutive and induced levels of heat shock proteins (HSP) and ability to develop thermotolerance.

Reflecting their chemosensitivity *in vivo*, the testis cancer cells are hypersensitive to chemotherapeutic drugs (Masters, J.R.W. *et al. IJC*, **53**, 340; 1993). Comparing their sensitivity to heat shock, the percentage clonogenic cell survival for the bladder lines ranged from 51.6–69.3% in the bladder lines compared to only 0.8–15.6% in the testis lines following a 30 min exposure to 45°C. Constitutive levels of HSP70 (72 and 73) and HSP90 showed no association with heat sensitivity, but HSP27 was virtually undetectable (Western blotting/scanning densitometry) in testis cancer cells, in contrast to the high levels detected in bladder cancer cells. Following an equitoxic heat shock, HSP72 synthesis continued for 10 h in the bladder line HT1376 (45°C/60 min) compared to only 4 h in the testis line 833K (45°C/15 min). Thermotolerance also differed between the two cell types. Following a priming heat shock at 42°C, thermotolerance developed more rapidly and was prolonged in the bladder line HT1376 compared to the testis line 833K.

In conclusion, testis tumour cells are hypersensitive to heat shock, are less able to develop thermotolerance and express HSPs, and contain low levels of HSP27.

### 3.10 Cyclophosphamide pharmacokinetics in children: sources of inter-patient variation

S.M. Yule[1], A.V. Boddy[2], M. Cole[3], L. Price[1], R. Wyllie[1], A.D.J. Pearson[1] & J.R. Idle[2]

*Departments of [1]Child Health, [2]Pharmacological Sciences and [3]Medical Statistics, The Medical School, Newcastle upon Tyne NE2 4HH, UK.*

Cyclophosphamide (CP) remains widely used in the treatment of paediatric malignancy. Hepatic metabolism of CP is necessary to produce the active alkylating species phosphoramide mustard.

Several authors have demonstrated considerable inter-patient variability in CP pharmacokinetics in adults with a single study linking these differences to the results of treatment. Cyclophosphamide kinetics were measured in 33 children (age 2 months–18 years). Each child received the drug as a one hour infusion (dose 0.37–2.49 $g/m^2$). Plasma levels of CP were measured using a quantitative Thin Layer Chromatography technique. The plasma half-life ($t_{1/2}$), Volume of Distribution ($V_d$) and Clearance (Cl) were obtained from a one compartment model with first order elimination kinetics.

Considerable inter-patient variation was seen. Cyclophosphamide $t_{1/2}$ exhibited 14-fold variation with, seven-fold variation observed in Cl and four-fold variation in $V_d$.

Cyclophosphamide $t_{1/2}$ increased with age, dose ($mg/m^2$), and concurrent administration of allopurinol. The $t_{1/2}$ in children receiving repeated courses of CP was shorter than those receiving the drug for the first time. Children who received dexamethasone prior to the study exhibited an increased Cl.

This study documents the extent of inter-patient variation amongst children with cancer. The prolonged $t_{1/2}$ seen at higher doses may reflect saturation of hepatic metabolism. Other differences may be the result of inherited variation in cytochrome P450-linked enyzme activity. The influence of allopurinol and dexamethasone may be due to hepatic enzyme inhibition and induction respectively. This study suggests that concurrent medication may alter CP pharmacokinetics and thus therapeutic effect.

### 4.1 Phosphorylation of the human oestrogen receptor

S. Ali[1], P. Pace[1], D. Metzger[2], P. Chambon[2] & R.C. Coombes[1]

*[1]Department of Medical Oncology, Charing Cross Hospital, London W6 8RF, UK; [2]LGME-CNRS, U184 de l'INSERM, Faculte de Medicine, 11 Rue Humann, 67085 Strasbourg.*

The importance of the oestrogen receptor (ER) in breast cancer is well established. Molecular cloning and functional analyses have shown that the ER is a ligand-inducible transcription factor which activates transcription by binding to short DNA palindromic elements in promoters of responsive genes.

We (and others) have recently shown that the ER is phosphorylated upon binding oestradiol. We have further shown that the human ER is phosphorylated on 4–5 sites in the presence of oestradiol and that the anti-oestrogens tamoxifen and ICI 164,384 also induce ER phosphorylation, although at much lower levels than oestradiol. Phosphoamino-acid analysis shows that most of these sites are serine residues. Using deletional analysis we have mapped the N-terminal-most phosphorylation site to serine 118. The region (region A/B), containing serine 118 is required for correct promoter and tissue-specific trans-activation by human ER and mutation of serine 118 drastically reduces *trans*-activation by human ER, but has no effect on DNA or hormone binding by the ER.

We have subsequently also shown that the ER can be phosphorylated *in vitro* by at least three protein kinases involving different signal transduction pathways.

These results and their importance for the regulation of ER function will be discussed.

### 4.2 Basic fibroblast growth factor-binding proteoglycans of human breast

P.J. Browne[1], J.J. Gomm[1], R.C. Coope[1], R.C. Coombes[1] & R.M. Mason[3]

[1]*Department of Medical Oncology and* [2]*Department of Biochemistry, Charing Cross and Westminister Medical School, London W6 8RP, UK.*

In the normal human breast basic fibroblast growth factor (bfgf) is concentrated in the myo-epithelial cell layer. To further define the role of bfgf in breast growth and development, we measured the ability of proteoglycans (PGs) produced by normal and malignant human breast cells to bind bfgf. Human recombinant bfgf was linked to a solid support, packed into a mini-column and the elution profiles of $^{35}$S-labelled PGs in a stepwise NaCl concentration gradient were determined. The malignant breast cell line, MCF-7 and the normal, immortalised breast cell lines, HBL-100, MCF-10A and MTSV1-7 were all found to express bfgf-binding PGs with a similar range of affinities. The PGs derived from the culture medium contained a greater proportion of higher affinity molecules than the cell-associated PGs. To investigate normal breast cells, reduction mammoplasty tissue was digested with collagenase following which pure populations of epithelial and myo-epithelial cells were prepared from trypsin-disaggregated organoids by immunomagnetic sorting using the Epithelial Membrane Antigen (EMA) for epithelial cells and the CD10 marker for myo-epithelial cells. Cultures were also established from the collagenase digests by selecting those cells capable of adhering rapidly to a plastic substrate. These cultures, designated stromal, had a fibroblastic morphology and were negative for EMA, cytokeratins 14 and 18 and collagen IV. The stromal cultures grew extremely well while the epithelial and myo-epithelial cultures proliferated more slowly and died out after 4–5 weeks. We have demonstrated bfgf-binding PGs in stromal and epithelial cell cultures but, to date, we have been unable to do so with myo-epithelial cultures. We hope to overcome this problem by modifying the immunomagnetic sorting process and/or the culture medium.

### 4.3 Expression of variant fibroblast growth factor receptors in human breast

Y.A. Luqmani, C. Mortimer, G. Bansal, L. Buluwela & R.C. Coombes

*Department of Medical Oncology, Charing Cross Hospital, London, UK.*

The widely distributed super family of heparin binding FGF's influence a diverse range of cellular activities exerted through specific cell surface receptors, for which at least four separate genes have been cloned: all of these include three extracellular Ig-like loops, a transmembrane region and a split cytoplasmic tyrosine kinase encoded domain. Several alternatively spliced mRNA isoforms have been identified. We have studied (using PCR) the expression, in RNA extracted from a large number of primary breast cancers, normal breast and cell lines, of two such variants affecting the extracellular Ig-like region. Both two loop and three loop forms of FGFR1 were found in all samples, with the former as the predominant type in cancers as compared to normal breast ($p < 0.02$): also reflected in the cell lines. Patients with high 2/3 loop tumour ratios had reduced relapse free survival ($p < 0.007$) particularly in the ER negative sub-group. Alternative usage of an exon loop results in the described BEK and SAM variants of FGFR2, both of which were found to be present in all tissues examined. SAM was predominantly expressed in cell lines of epithelial lineage, and only 4/32 (including two breast) lines expressed both variants significantly. Ligand specificity between these forms may be implicated in paracrine regulatory mechanisms involving for example, interaction with stromal derived KGF which binds only the SAM variant.

### 4.4 mRNA localisation for FGF's and their receptors in resected adenocarcinomas of the human pancreas

H.Y. Leung[1,2], C.M. Hughes[1], G. Klöppel[3], R.C.N. Williamson[2] & N.R. Lemoine[1]

[1]*ICRF Oncology Group,* [2]*Department of Surgery, Hammersmith Hospital, London, UK;* [3]*Academic Hospital Jette, Laarbeeklaan 101, Brussels.*

Using a panel of 14 human pancreatic cancer cell lines, we have previously identified a characteristic pattern of expression for the first seven members of Fibroblast Growth Factors and their four receptors. We now report our observations from *in situ* hybridization (ISH) examination on a collection of archival paraffin embedded blocks of human pancreatic cancer. The non-radioactive digoxigenin system is used to generate specific antisense orientated riboprobes for FGF1 and FGF2, and the four FGFRs (FGFR1, 2, 3 and 4). The corresponding sense strand riboprobes were used as controls. Sections from the blocks were processed according to standard ISH protocol and signals were generated with alkaline phosphatase conjugated anti-digoxigenin antibody.

We confirmed the expression of both FGF/FGFR by tumour cells, with the presence of a potential autocrine loop activity in 46% of the cases studied. FGF2 and FGFR3 were the most commonly expressed ligand and receptor (46% and 76% respectively). The acinar cells were found to have a heterogeneous expression pattern for FGFRs while duct cells, islet cells and stromal components were negative.

In summary we report an additional piece of evidence strongly suggesting an important role of FGFs and their receptors in human pancreatic adenocarcinoma.

### 4.5 Differing behaviour of colon carcinoma cells and correlation with epidermal growth factor receptor ligand expression

N. Solic, S.J. Holt, A. Richter, J. Collins, R. Adam, P. Alexander & D.E. Davies

*CRC Medical Oncology Unit, Southampton General Hospital, Southampton SO9 4XY, UK.*

Recent studies (Saeki, T., Stromberg, K., Qi, C-F. (1992). *Cancer Research*, **52,** 3467) have reported expression of amphiregulin (AR) in normal colon and well differentiated colonic tumours whereas transforming growth factor alpha (TGFα) expression is found in poorly differentiated tumours. These results suggest that differential expression of epidermal growth factor receptor (EGF-R) ligands may be associated with progression from premalignant to malignant carcinoma of the colon.

We have investigated EGF-R ligand expression and their contribution to the growth and differentiation of colon carcinoma cells (GP cells) which we have recently established in culture. Within the cell culture, two distinct sub-types of cells with differing morphologies could be identified. One group of cells tended to form multilayered colonies whereas the other group was more fibroblastic in appearance. This morphological difference persisted when individual cells were cloned. Two of these clones, identified as GP5d (multilayered) and GP2d (fibroblastic), were shown to have identical karyotypes, both possessing a deletion on the long arm of chromosome 5. Both clones grew as xenografts in nude mice, GP2d formed a well differentiated tumour while GP5d formed an undifferentiated invasive tumour. The clones were shown by RT-PCR and immunocytochemical staining to express three ligands for EGF-R, namely AR, TGF$\alpha$ and heparin-binding EGF, as well as the receptor itself. Whereas AR expression was comparable in the two clones, GP5d expressed a higher level of TGF$\alpha$. Although proliferation of the cells in serum free medium was slightly enhanced in the presence of EGF (1–25 ng/ml), by far the most striking effect of this growth factor was on the morphology of the cells causing them to spread and become more flattened in appearance. This change was shown to be associated with a redistribution of desmosomal proteins. The effect was also observed using TGF$\alpha$ but not using comparable doses of recombinant amphiregulin (as evaluated in a standard mitogenesis assay using NR6/HER fibroblasts).

The availability of cells derived from the same tumour with persistent differences in behaviour may provide a useful experimental model for the further study of oncogenes and/or transcription factors in colon carcinoma and may provide new insight into factors controlling progression of this disease.

### 5.1 Correlation of EGF-induced protein tyrosine-phosphorylation with loss of tumour cell adhesion *in vitro*

T. Gulliford & R.J. Epstein

*CRC Laboratories, Department of Medical Oncology, Charing Cross Hospital, London W6 8RF, UK.*

Human cancer metastasis displays 'soil-and-seed' characteristics which may reflect tumour cell expression of specific cytokines and/or adhesion molecules. Here we report that A431 human squamous carcinoma cells undergo striking morphologic and adhesive changes *in vitro* within 5 min of exposure to epidermal growth factor (EGF). Loss of cell adhesion closely parallels the dose- and time-dependency of EGF receptor autophosphorylation. Equally rapid morphologic effects are apparent as increased cell 'rounding-up' following trypsinisation, consistent with EGF-induced tyrosine phosphorylation of cytoskeletal-associated substrates. Similar *in vitro* variations of cell adhesion are seen in tumour cell lines expressing *erb*B-2 receptors with different basal activities. Exposure of EGF-deprived A431 cells to the tyrosine phosphatase inhibitor sodium orthovanadate (1 mM for 1 h) does not cause receptor tyrosine phosphorylation, indicating that phosphatase activation in these cells requires EGF-induced tyrosine phosphorylation. Since the extracellular domains of transmembrane tyrosine phosphatases contain immunoglobulin-like and/or fibronectin-like motifs which promote cell adhesion when expressed in catalytically inert mutants (Gebbink *et al.*, *J. Biol. Chem.*, 1993, **268**, 16101), our findings raise the interesting possibility that growth factor-induced activation of transmembrane phosphatases may abrogate tumour cell adhesion *in vivo*.

### 5.2 Pattern of adhesion molecule expression on bladder tumour biopsy and tumour cell lines: effectiveness of cytokine stimulation in upregulation of these molecules

A.M.E. Nouri, R.F. Hussain & R.T.D. Oliver

*Department of Medical Oncology, The Royal London Hospital, London E1, UK.*

The pattern of cell adhesion molecules (CAM) i.e. leukocyte function associated antigen-3 (LFA-3) and intercellular adhesion molecule-1 (ICAM-1) expression on bladder tumour biopsies and tumour cell lines was investigated using different techniques.

The results showed that 15 of the 25 tumour biopsies were negative for ICAM-1 and amongst the remaining cases, only one showed strong staining whereas, LFA-3 was expressed on 21 of 23 tumours tested. The pattern of ICAM-1 expression on tumour cell lines was very different from tumour biopsies in that there were many more strongly expressing tumours. In the case of LFA-3, whilst the results of established cell lines were very similar to those of tumour biopsies, there were no strongly expressing cases among all the seven primary lines studied, indicating that the *in vitro* tumour lines may not always be the true representative of the original tumour.

The comparison between ICAM-1 and class II antigen expression on tumour biopsies showed that there were 11 of 18 cases where either both these molecules were expressed together or were completely absent. In the remaining seven, there were six cases where only strong class II expression was observed.

Exposure of established cell lines to cytokines showed that interferons (IFN) $\alpha$ had no effect on either ICAM-1 or LFA-3 expression. Similarly, IFN$\gamma$ showed no effect on LFA-3 molecule. However, it induced ICAM-1 on eight of 11 lines investigated, under conditions where IFN$\alpha$ failed to have any significant activity. The mean $\pm$ SD for control value of eight responder cell lines was $617 \pm 406$ and following IFN$\alpha$ and IFN$\gamma$ stimulation the values were $702 \pm 563$ ($p = 0.022$) and $943 \pm 471$ ($p = 0.001$) cpm respectively under conditions where established cell lines treated with these two cytokines showed a similar increase in the susceptibility to non-MHC-restricted cytotoxicity. Transfection of b2-m gene to correct defective class 1 antigen on a cell line had no effect on class 11 and ICAM-1 expression.

These results indicate that there is a significant minority of bladder tumours with defective CAM and this could be an important factor in the overall tumour escape mechanism(s).

### 5.3 Inhibition of heterotypic cell to cell adhesion by an RGD containing peptide

R.D. Bliss, J.A. Kirby, D.A. Browell & T.W.J. Lennard

*Department of Surgery, University of Newcastle upon Tyne, UK.*

Binding of tumour cells to the vascular endothelium and the extracellular matrix is essential to the metastatic cascade. Blockade of this process has been shown to reduce metastases *in vivo* and *in vitro*. Integrins are known to be involved in this process. Many Integrins bind to an RGD motif which is common to many of their ligands.

The function of this motif was examined by measuring the adhesion of MCF-7 cells, a breast tumour cell line, to EAhy926, an endothelial hybridoma, using varying concentrations of two different peptides.

Confluent monolayers of EAhy926 were grown on 96-well micro-titre plates and differing concentrations of either the RGD or RGDS peptides were added. MCF-7 cells were labelled with $^{51}Cr$ and $3 \times 10^4$ cells added to the endothelial monolayer. After 2 h, non-adherent cells were removed and the remaining bound cells were lysed. The $^{51}Cr$ release was then measured using a gamma-spectrometer.

This showed that cell–cell adhesion is significantly inhibited experimentally by use of an RGD-containing peptide, but not by the tripeptide itself.

| Peptide | *Inhibition of adhesion (%)[1] produced by varying concentrations of peptide* 0.1 mg/ml | 10 µg/ml | 1 µg/ml | 0.1 µg/ml | 10 ng/ml | 1 ng/ml |
|---|---|---|---|---|---|---|
| RGD | 3.3 | −16.6 | −12.4 | −5.4 | −2.9 | ND |
| RGDS | 25.8** | 23.8* | 22.9* | 17.5 | 9.6 | 10.9 |

[1]Mean (n = 26); **p<0.005; *p<0.05

We conclude that Integrin blockade by an appropriate peptide can inhibit tumour cell adhesion to vascular endothelium; this may reduce the incidence of metastases.

### 5.4 Inhibition of colon cancer cell motility and attachment to ECM by interleukin-12

S. Hiscox, M.B. Hallett, M.C.A. Puntis & W.G. Jiang

*Department of Surgery, University of Wales College of Medicine, Cardiff, UK.*

Both motility and attachment of tumour cells are regulated by a number of cytokines, including scatter factor (SF). In this study we investigated the effects of interleukin 12 (IL-12, also known as natural killer cell stimulatory factor, NKSF), on the motility and attachment of human colon cancer cells HRT18 and HT115.

The MTT assay was used to quantify attachment to the reconstituted basement membrane, Matrigel. The effect on motility induced by SF (10 ng/ml) was determined by measuring the dissociation of the cells from micro-carrier beads. Changes in cell-surface E-cadherin were also determined via indirect immunofluorescence and flow cytometry.

Recombinant human IL-2 (p40) was shown to inhibit attachment of colon cancer cells to Matrigel (% attachment = 77.0±7.1% for HRT18 and 62.2±5.0% for HT115 in control; 24.0±9.8% for HRT18 and 9.6±2.4% for HT115 with IL-12 (50 ng/ml)). Scatter factor-induced motility was decreased by IL-12 as shown by the dissociation assay (results shown as motility index (mean±sem)).

| | *Control* | *+SF* | *+SF+IL-12* |
|---|---|---|---|
| HRT18 | 1.00±0.52 | 3.26±0.58 | 1.34±0.32 |
| HT115 | 1.00±0.03 | 3.00±0.78 | 1.96±0.33 |

Interleukin-12 induced effects occurred in a concentration dependent manner (2–200 ng/ml) and were blocked by an antibody specific for the IL-12 ligand. Data obtained by flow cytometry indicated up-regulation of cell-surface E-cadherin.

It is proposed that IL-12 inhibits cell motility and attachment to extracellular matrix proteins via the up-regulation of the cell-surface E-cadherin.

### 5.5 Swainsonine directly inhibits invasion of human colorectal tumour cells *in vitro*

C. Galustian, S. Foulds & P.J. Guillou

*Academic Surgical Unit, St Mary's Hospital Medical School, London W2 1NY, UK.*

Swainsonine (SW), an inhibitor of glycoprotein processing, has been shown to inhibit metastasis of murine tumours *in vivo*. Previously, we have observed that one mechanism of this effect is an enhancement of anti-tumour immune functions. We now show that SW also has a direct inhibitory effect on basement membrane invasion of a human colorectal cell-line, SW620, *in vitro*. SW620 cells were incubated with varying SW concentrations for 4 days and then plated onto basement membrane matrigel coated filters in double chamber cassettes. A 5 h membrane invasion chamber system (MICS) assay was then performed. Tumour cells invading from upper to lower chambers were stained and counted. The results are shown below as % of control tumour invasion±s.e.

| *[SW](µg/ml)* | *0* | *1* | *2* | *5* | *10* |
|---|---|---|---|---|---|
| % invasion | 100±3.0 | 48±4.9* | 48±5.3* | 40±3.0* | 38±2.6* |

(p<0.01 by anova compared to control, * = p<0.01 by Duncan's test).

Mechanisms of this inhibition were then investigated. SW620 tumour cells incubated with 10 µg/ml SW for 4 days were 27.5% less adherent to basement membrane matrigel after 1 h than control cells (p<0.01 by unpaired t-test). SW also increased cell-cell aggregation in SW620 tumour cell cultures: SW620 cells pre-incubated with 10 µg/ml SW had 39%±6.3 of cell aggregates in the culture compared to 17.32%±5.1 in the controls (± = s.d., p<0.01 by Mann–Whitney U, n = 7). We then investigated the effect of SW on the expression of E-cadherin, a cell-cell adhesion molecule whose loss from cells is implicated in increased metastatic potential and incubated with fluorescein labelled anti-E-cadherin antibodies and cell-expression was increased from 18% in untreated cells to 25%±1.6 (±s.e.d. p<0.001) in cells treated with 10 µg/ml SW.

In summary, SW directly inhibits basement membrane invasion of colon tumour cells *in vitro*, and this effect may be related to changes in cell-matrix and cell-cell adhesion. These studies show further evidence of the potential of SW in anti-metastatic therapy.